# An Overview of the Genetics of *ABCA4* Retinopathies, an Evolving Story

**DOI:** 10.3390/genes12081241

**Published:** 2021-08-13

**Authors:** Saoud Al-Khuzaei, Suzanne Broadgate, Charlotte R. Foster, Mital Shah, Jing Yu, Susan M. Downes, Stephanie Halford

**Affiliations:** 1 Oxford Eye Hospital, John Radcliffe Hospital, Oxford University Hospitals NHS Foundation Trust, Oxford OX3 9DU, UK; saoud.al-khuzaei@wadham.ox.ac.uk (S.A.-K.); mital.shah@nhs.net (M.S.); 2 Nuffield Laboratory of Ophthalmology, Nuffield Department of Clinical Neuroscience, University of Oxford, Level 6 John Radcliffe Hospital, Headley Way, Oxford OX3 9DU, UK; suzanne.broadgate@ndcn.ox.ac.uk (S.B.); jing.yu@ndcn.ox.ac.uk (J.Y.); 3 Pathlab, 829 Cameron Road, Tauranga 3112, New Zealand; charlotte.foster@hotmail.com

**Keywords:** *ABCA4*, Stargardt disease, genetic testing, ABCA4-associated retinopathies, phenocopies

## Abstract

Stargardt disease (STGD1) and *ABCA4* retinopathies (ABCA4R) are caused by pathogenic variants in the *ABCA4* gene inherited in an autosomal recessive manner. The gene encodes an importer flippase protein that prevents the build-up of vitamin A derivatives that are toxic to the RPE. Diagnosing ABCA4R is complex due to its phenotypic variability and the presence of other inherited retinal dystrophy phenocopies. *ABCA4* is a large gene, comprising 50 exons; to date > 2000 variants have been described. These include missense, nonsense, splicing, structural, and deep intronic variants. Missense variants account for the majority of variants in ABCA4. However, in a significant proportion of patients with an ABCA4R phenotype, a second variant in ABCA4 is not identified. This could be due to the presence of yet unknown variants, or hypomorphic alleles being incorrectly classified as benign, or the possibility that the disease is caused by a variant in another gene. This underlines the importance of accurate genetic testing. The pathogenicity of novel variants can be predicted using in silico programs, but these rely on databases that are not ethnically diverse, thus highlighting the need for studies in differing populations. Functional studies in vitro are useful towards assessing protein function but do not directly measure the flippase activity. Obtaining an accurate molecular diagnosis is becoming increasingly more important as targeted therapeutic options become available; these include pharmacological, gene-based, and cell replacement-based therapies. The aim of this review is to provide an update on the current status of genotyping in ABCA4 and the status of the therapeutic approaches being investigated.

## 1. Introduction

Stargardt disease (STGD1, OMIM# 248200), otherwise known as Stargardt macular dystrophy, juvenile macular degeneration, or fundus flavimaculatus, is one of the most common causes of inherited retinal diseases. It is estimated to have an incidence of between 1 in 8000 and 1 in 10,000 [1]. STGD1 is normally detected in late childhood or early adulthood and is progressive, varies in severity, and sometimes vision loss may not be noticed until later in adulthood. It is inherited in an autosomal recessive manner and is caused by variants in the *ABCA4* gene [2], with a carrier frequency reported to be as high as 1 in 20 (depending on the population) [3,4].

**Figure 1:**
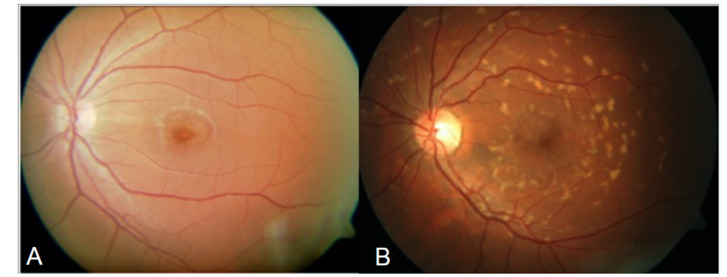
Colour fundus photographs: (**A**) foveal atrophy surrounded by a minimal amount of flecks; and (**B**) widespread flecks that would be consistent with a fundus flavimaculatus phenotype.

The disease was first described by Karl Stargardt in 1909 in two families with macular dystrophy associated with yellow-white pisciform flecks [5]. In 1965, Franceschetti used the term fundus flavimaculatus (FFM) to describe the widespread presence of flecks (Figure 1) [6,7].

**Figure 2:**
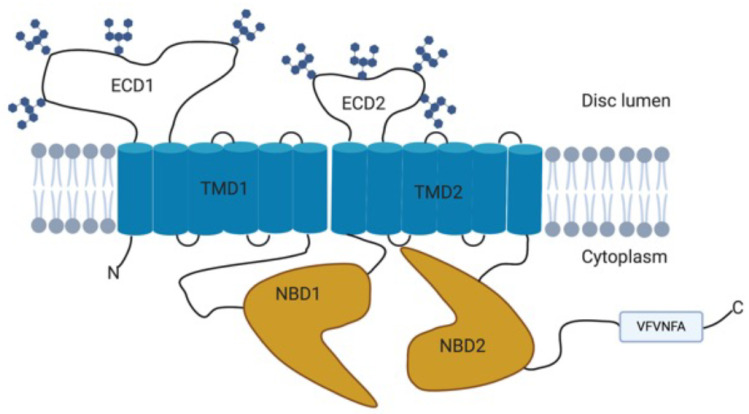
Schematic diagram of the structure of the ABCA4 protein showing the two transmembrane domains (TMD), the nucleotide-binding domains (NBD), and exocytoplasmic domains (ECD) that contain N-linked oligosaccharide chains and the C-terminal VFVNFA motif. Adapted from [33,37,38]. Created with BioRender.com (accessed on 1 June 2021).

**Figure 3:**
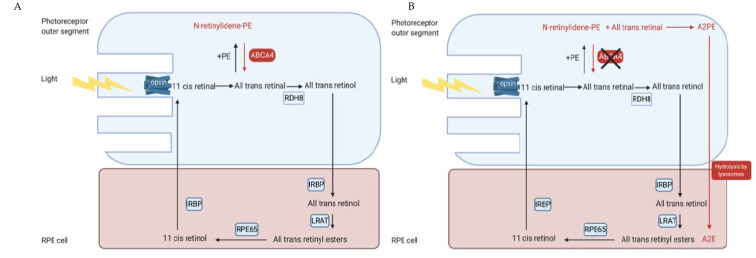
Schematic diagram illustrating the visual cycle in the photoreceptor outer segments and the RPE. (**A**) Light photobleaches the opsin and isomerises the 11-*cis*-retinal to ATR. Some ATR reversibly reacts with PE to form NrPE, which is flipped onto the cytoplasmic side by the ABCA4 protein. The NrPE is then hydrolysed to PE and ATR, thus preventing the accumulation of ATR on the luminal side. The ATR is then reduced to all-*trans*-retinol by RDH8 and then transported to the RPE cell by IRBP. In the RPE, the all-*trans*-retinol is esterified to all-*trans*-retinyl esters by LRAT, which is then converted to 11-*cis*-retinol by RPE65 isomerohydrolase and then oxidized to 11-*cis*-retinal by RDH and transported back to the photoreceptors by IRBP. (**B**) Schematic diagram illustrating the visual cycle in the presence of ABCA4 dysfunction. Dysfunction of the ABCA4 protein prevents the flipping of the NrPE from the luminal side to the cytoplasmic side of the photoreceptor outer segments, meaning that the NrPE accumulates and condenses with all-*trans*-retinal into A2PE. The photoreceptor outer segments are then shed and phagocytosed by the RPE cell, which then hydrolyse the A2PE to A2E [56]. Created with BioRender.com (accessed on 1 June 2021). Abbreviations: ATR: All-*trans*-retinal; A2E: *N*-retinyl-*N*-retinylidene ethanolamine; A2PE phosphatidyl-pyridinium bisretinoid; IRBP: inter photoreceptor binding protein; LRAT: lecithin retinol acyltransferase, NrPE: N-retinylidene phosphatidylethanolamine; PE: phosphatidylethanolamine; RDH8: retinol dehydrogenase 8; RPE: retinal pigment epithelial.

Genetic linkage in the early 1990s localised STGD1 and FFM to the same locus on the short arm of chromosome 1, 1p13–p21 [8], thus confirming that the two conditions were part of the same disease spectrum [9,10,11]. This genetic locus was subsequently refined to a 2-cM interval on 1p21–22.1 [12]. In 1997, Allikmets et al. demonstrated that the disease was caused by variants in the *ABCA4* (ATP-binding cassette transporter, alpha 4 subunit) gene, originally called *ABCR* [2]. The *ABCA4* transcript was first detected in rod photoreceptors [2,13,14] and then in cones [15]. The *ABCA4* gene encodes a transmembrane protein that is localised to the rim of the disc membranes in the outer segments of rod and cone photoreceptor cells and plays an essential role in retinoid recycling in the visual cycle [13,15] (see Section 2.1 and Section 2.2 and Figures 2 and 3).

Stargardt disease has a highly variable phenotype but there are three characteristic features: the presence of flecks, macular atrophy, and sparing of the peripapillary region, which if seen together are indicative of a retinal disorder associated with variants in *ABCA4*. [16,17,18]. Cideciyan et al. used the term *ABCA4*-associated retinal degenerations in 2005 [17] and since then other authors have used the term *ABCA4*-associated retinopathies [19,20,21]; for the purposes of this review, ABCA4R will be used to denote *ABCA4* retinopathies. The progression of the disease is also variable but patients with early childhood onset typically have a more severe phenotype and more rapid disease progression [22,23,24,25]. In contrast, patients with a late onset disease (>45 years of age) usually have a milder phenotype and slower progression [26,27,28,29]. Information regarding the imaging and characterisation of the different *ABCA4* retinopathies (ABCA4R) is described in a review [30]. 

Retinal disorders with clinical phenotypes resembling STGD1 but with a dominant pattern of inheritance are referred to as “Stargardt like” and have been assigned to STGD2–4 [31,32]. There are other retinal conditions associated with different gene variants that mimic the STGD1 phenotype, known as phenocopies, which can complicate a diagnosis and underline the need for accurate genetic testing (see Section 3).

The aim of this review article is to give an overview of the current status of genotyping in *ABCA4*, an update on missing heritability in *ABCA4,* phenocopies, the effect of genotype on the severity of the phenotype, and assessment techniques to predict the functional consequences of the variants. We will also provide an update on the current therapeutic approaches that are being investigated. 

## 2. Function of ABCA4

### 2.1. Structure 

The *ABCA4* gene comprises 50 exons, spanning 128 kb of genomic DNA, and encodes a 2273 amino acid protein with a molecular mass of approximately 250 kDa [2,33]. The ABCA4 protein is a member of the ATP-binding cassette (ABC) transporter superfamily belonging to the A subfamily [2]. Structurally, the ABCA4 protein consists of two non-identical but homologous parts each of which contain a transmembrane domain (TMD) with six membrane spanning helices, a nucleotide-binding domain (NBD), and an exocytoplasmic domain (ECD) (Figure 2) [34,35,36].

### 2.2. Function and Role within the Visual Cycle

**Figure 4:**
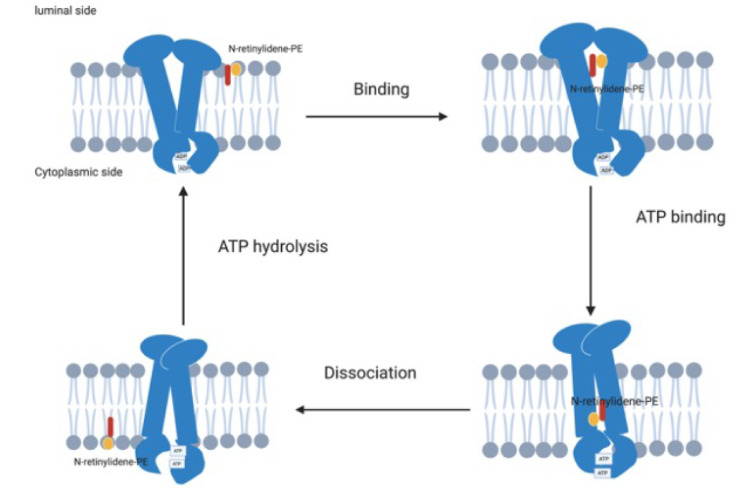
Schematic diagram of the ABCA4 protein actively transporting NrPE from the luminal side of the photoreceptor disc membrane to the cytoplasmic side. The ADP is initially bound to the NBD and the ABCA4 binds the NrPE on the luminal side of the photoreceptor disc membrane; this is followed by binding of ATP to the NBDs, leading to a conformational change that creates a low affinity binding site on the cytoplasmic side, resulting in dissociation of the NrPE from the ABCA4 protein and followed by hydrolysis of the ATP, returning the ABCA4 to its primary conformation. Adapted from Molday et al. [37]. Created with BioRender.com (accessed on 1 June 2021). Abbreviations: NBD: nuclear binding domain; NrPE: N-retinylidene phosphatidylethanolamine.

In the visual cycle, light isomerises the chromophore 11-*cis*-retinal to all-*trans*-retinal, which is then released from the binding pocket of the rod or cone opsin. The majority of all-*trans*-retinal is reduced to all-*trans*-retinol by retinol dehydrogenase 8 (RDH8) [39] and transferred to the retinal pigment epithelium (RPE) by interphotoreceptor retinoid binding protein (IRBP). It is then esterified by lecithin retinol acyltransferase (LRAT) and converted to 11-*cis*-retinol by RPE65 isomerohydrolase. The 11-*cis*-retinol is oxidised to 11-*cis*-retinal by RDH8 and transported back to the photoreceptors by IRBP where it rebinds to the rod and cone opsin to be re-used in the visual cycle [35,37,40,41] (Figure 3a,b). However, some of the all-*trans*-retinal reversibly reacts with phosphatidylethanolamine (PE) to form N-retinylidene phosphatidylethanolamine (NrPE) in the photoreceptor disc membrane [42,43]. In the normal visual cycle, the function of ABCA4 is to import the ABC proteins NrPE [44,45] and PE [45]. This is unique to ABCA4 as it is the only known ABC protein that functions as an importer in mammals [45]. The ABCA4 protein flips the NrPE from the luminal side to the cytoplasmic side of the photoreceptor disc membrane (Figure 4) where the NrPE is subsequently hydrolysed to PE and all-*trans*-retinal. The all-*trans*-retinal is then reduced to all-*trans*-retinol by RDH8 in order to resynthesize 11-*cis*-retinal, which then re-enters the visual cycle [33,35,37,46].

Variants in *ABCA4* can affect the structure and function of the ABCA4 protein. Dysfunction of the ABCA4 protein leads to an accumulation of NrPE and all-*trans*-retinal within the photoreceptor disc membrane, which condenses to form phosphatidyl-pyridinium bisretinoid (A2PE) [47]. The photoreceptor outer-segments are then shed and phagocytosed by RPE and the A2PE is hydrolysed by lysosomal enzymes to bisretinoid *N*-retinyl-*N*-retinylidene ethanolamine (A2E) [48], which cannot be metabolised further [49,50]. As a result, the A2E accumulates within the RPE and forms a major component of lipofuscin, which is toxic to the RPE, thus leading to degeneration of the RPE and subsequently loss of the photoreceptor cells [33,35,46,50,51] (Figure 3B). Recently, *ABCA4* has also been linked with decreasing excess 11-*cis*-retinal, which also reversibly reacts with PE to form N-11-*cis*-retinylidene-phosphatidylethanolamine, which, in turn, is also transported by the ABCA4 protein, meaning that accumulation of 11-*cis* retinal might also be linked with STGD1 [52].

Genotype–phenotype correlation in *ABCA4* is generally thought to correlate with the remaining function of ABCA4, meaning that more severe combinations, such as having two null variants, results in a severe phenotype with an early disease onset whilst milder variants are linked with later onset [53,54,55]. In this review, we will describe the effects of these different variants and the up-to-date methods used to investigate a variant’s severity.

## 3. Clinical Phenotypes and Phenocopies in *ABCA4* Retinopathies (ABCA4R)

The phenotype in *ABCA4* retinopathies (ABCA4R) is highly variable. The terms STGD1/FFM refer to the typical STGD1 and fundus flavimaculatus phenotype characterised by flecks and macular atrophy. For this publication, the term ABCA4R will be used for the wide range of phenotypes previously reported in association with variants in *ABCA4*. These include bull’s eye maculopathy (BEM) [57,58], retinitis pigmentosa with bone spicule pigmentary deposition [59,60], cone rod dystrophy [61], choriocapillaris dystrophy [62], and rapid onset chorioretinopathy [63]. 

Diagnosing ABCA4R is further complicated by phenotypic overlap with other disorders that share similar features (see Table 1). These may be referred to using terms such as Stargardt like or pseudo-fundus flavimaculatus. In 1994, the term “Stargardt like” was used to describe a dominantly inherited macular dystrophy located on chromosome 6q [31]. The STGD3 (OMIM# 600110) type was subsequently shown to be caused by variants in *ELOVL4* [64]. The STGD4 (OMIM# 603786) locus was mapped to chromosome 4 [32] and is linked to dominant *PROM1* variants [65]. STGD2, which was originally mapped to a locus on chromosome 13q34 [66], was subsequently shown to be caused by *ELOV4* variants as in STGD3 [64]. The phenotype in STGD3 includes early onset disease with pigmentary changes and flecks within the macular region [67]. For STGD4, changes in *PROM1* have been associated with variable phenotypes, which include cone rod dystrophy, macular dystrophy, retinitis pigmentosa [68], BEM [69], and the presence of flecks [32].

The term “phenocopies” is used for IRDs that manifest with a similar phenotype but are caused by variants in different genes. For ABCA4R, the commonest phenocopy is the pattern dystrophy caused by variants in *PRPH2* inherited in an autosomal dominant manner [70]. Indeed, Ibanez et al. recently found that a *PRPH2* variant was identified in 10% of their misdiagnosed STGD1 patients [71]. The typical phenotype linked to *PRPH2* is a late-onset pattern dystrophy with variable penetrance within families. However, variants in *PRPH2* have also been associated with the characteristic features of STGD1, such as flecks, macular atrophy, and changes in a full-field electroretinogram (ffERG) [70]. Occasionally, the peripapillary region can be spared, as in STGD1 [78]. Heterozygous variants in *CRX* can result in a phenotype that mimics STGD1 and is associated with late-onset disease with a bull’s eye maculopathy (BEM) [72], and cone and rod dysfunction detected on electrodiagnostic testing [73]. In a recent study, Wolock et al. found that *CRX* and *PRPH2* variants accounted for ~10% of their “STGD1” patients not found to carry *ABCA4* variants. *CDH3* can also be misdiagnosed as STGD1 due to the juvenile onset macular dystrophy, but these patients can be distinguished by the presence of hypotrichosis of scalp hair [76,77]. 

*BEST1* can also be misdiagnosed as STGD1 in compound heterozygous patients with autosomal recessive variants due to the widespread presence of vitelliform deposits that can be confused for flecks, particularly in autosomal recessive bestrophinopathies [74]. Distinguishing autosomal dominant Best disease (BEST1) is usually easy as it is characterised in most cases by the presence of a solitary yellow deposit usually at the macula, which is round and far larger than a fleck. In addition, the presence of an abnormal electrooculogram (EOG) and a normal electroretinogram (ERG) is discriminatory. However, autosomal recessive bestrophinopathy may be misdiagnosed as an ABCA4R due to the presence of multiple vitelliform deposits, but the shape and distribution of these deposits is different from STGD1, and macular schitic changes are often visible in autosomal recessive bestrophinopathy [74]. Genetic testing results are reassuring when differentiating the two conditions [75]. Another misdiagnosis of STGD1 can include cone rod dystrophy (CRD).

Toxicity from drugs may cause a BEM phenotype. BEM is observed in susceptible patients who develop a toxic retinopathy in response to quinolones, such as hydroxychloroquine and chloroquine [79]. Other drugs have been associated with BEM, but less frequently; an overview of these can be found in a review by Mitra et al. [80].

Another compounding factor is the high carrier frequency of variants in *ABCA4* observed in the general population. This can result in a pseudo-dominant inheritance pattern being observed. As seen when the child and one parent is affected but the other parent is an unaffected carrier, the disease appears to be inherited dominantly. Variability is also observed within families as a result of individuals carrying a different combination of variants. Of significant interest is the observation that siblings carrying the same variants may have different phenotypes. It has been hypothesised that this could be due to genetic modifiers or environmental factors, which could affect the severity and penetrance of the disease [81,82,83,84]. Indeed, Runhart et al. clearly proposed the presence of modifiers when they reported non-penetrance of null/severe variants when in *trans* with the c. p.(Asn1868Ile) variant [85] and also proposed a female sex bias for the p.(Asn1868Ile) and the c.5882G>A p.(Gly1961Glu) variants [86]. However, Lee et al. did not find a significant sex bias for either of these variants when they investigated their cohort of patients [87].

The variability in the phenotypes seen in ABCA4R and similarities in the phenotypes caused by other genes can sometimes make it difficult to make a definitive clinical diagnosis, even for an experienced clinician. This highlights the importance of genetic testing to provide a molecular diagnosis. The importance of this is twofold for patients with a Stargardt-like phenotype but with variants in genes other than *ABCA4*: (1) for correct genetic counselling regarding inheritance patterns; and (2) in order to prevent incorrect recruitment into therapeutic trials, with the risk of invasive procedures that could lead to harm and no therapeutic benefit. 

## 4. Genetics of ABCA4R

Since the gene was first identified by Allikmets et al. in 1997 [2], with a subsequent report of the full-length gene by Gerber et al. in 1998, genetic testing has been undertaken in many patient cohorts [88]. To date, more than 2000 *ABCA4* variants have been reported in the literature (www.lovd.nl/ABCA4 accessed on 1 June 2021).

### 4.1. Genetic Testing in ABCA4

Initially, single-strand conformation polymorphism (SSCP) analysis was used to screen the gene [2,53] but a poor detection rate led to the use of a variety of techniques, each of which had advantages and disadvantages. The development of an arrayed primer extension (APEX) array by Asper Biotech increased the detection rate but only previously reported variants were able to be detected as they were included in the initial probe design [4]. This meant that novel variants were not detected. Other techniques that can be used in conjunction with panels to improve the detection rates include denaturing gradient gel electrophoresis (DGGE), denaturing high-performance liquid chromatography (dHPLC), and high-resolution melting (HRM) [89,90,91]. Multiplex ligation-dependent probe amplification (MLPA) has also been used to detect deletions or duplications in the *ABCA4* gene [90]. Using Sanger sequencing for all 50 exons and at least 10 bp of flanking introns achieves a detection rate of up to 80% of alleles [3,92]. However, it is expensive and time consuming, which limits its use in large cohorts of patients.

More recently, next generation sequencing (NGS) has revolutionized genetic testing. Targeted exome, whole exome (WES), and whole genome sequencing (WGS) are highly automatable techniques that can be used to sequence large cohorts of patients and enable the detection of variants in both known IRD and novel genes [93,94]. WGS can detect both exonic and intronic variants [95]. However, the downside of these techniques is that they require large amounts of space for data storage and require expertise in bioinformatics (for a review, see Broadgate et al. (2017) [96]). A recent report using single-molecule molecular inversion probe (smMIP)-based sequencing has demonstrated a coverage of 97.4% of the 128 kb *ABCA4* gene, including all 50 exons and splice sites, and can also identify copy number variants [97]. The main benefits of smMIP-based sequencing are that it is cost effective and can be used to sequence large cohorts of patients [97], which makes it a valuable sequencing technique for conditions with a characteristic phenotype such as STGD1.

These advances in sequencing techniques have improved our ability to detect variants but *ABCA4* remains a complex gene due to its size, highly polymorphic nature, and wide spectrum of variants identified.

### 4.2. Spectrum of Pathogenic Variants in ABCA4

Sequencing carried out in large cohorts of patients has shown that *ABCA4* variants include missense, nonsense, frameshift, splice site, and structural variants. Several studies have shown that the majority of *ABCA4* variants reported to date are missense variants [98,99,100,101,102,103,104]. Missense variants are usually caused by a single nucleotide change, which usually results in a change in the amino acid. The consequences of this are difficult to predict as not all amino acid changes lead to changes in the protein structure that result in a change in function [54,105,106]. Many of these missense variants are rare, which further complicates determining their severity and whether they are truly pathogenic [20,107,108]. The effect of deleterious variants, such as nonsense or frameshift variants, are easier to predict as these variants create truncated proteins with no ABCA4 activity. The term structural variant refers to copy number variants, which encompass deletions, insertions, and duplications, as well as inversions and translocations [109]. These structural variants can lead to a significant alteration in protein structure [110]. Non-canonical splice site (NCSS) variants occur in intronic regions and the first and last nucleotide positions of exons and may alter the cryptic splice sites, thus activating them. These variants may result in abnormal splicing efficiency or alter the order of the splicing steps. Variants in these cryptic splice sites can produce a pseudoexon that frequently contains premature termination codons, thus resulting in nonsense-mediated decay (NMD) [111]. 

### 4.3. Variability in Variants between Populations 

The majority of studies on *ABCA4* have been conducted on patients of European descent. As a result, the variant databases have an inherent bias for this population. Indeed, Chinese and African-American patients have been found to have a higher prevalence of variants not seen in European patients and are also less likely to carry the variants frequently detected in this population [103,112]. Moreover, founder variants are often found in isolated populations and those originating from settlers. In these populations, the founder variants can explain a significant proportion of STGD1 with a high molecular diagnosis rate despite a small number of variants being detected in the population [113,114,115]. Detection rates for the *ABCA4* variants appear to be similar in populations (Table 2), although it is important to note that the detection rate is mostly influenced by the clinical expertise and how well defined the phenotype of the patient is. However, it is of note that the detection rate in French Canadian patients was very low at 33%, which suggests the presence of yet undiscovered founder variants in this population [116]. The differences in the frequencies and of the variants seen in different populations highlights the importance of accurate genetic testing to identify the causative variants and limitations of using panel-based technologies that might not detect variants that are commonly seen in a specific population.

The complexity in genetic testing in *ABCA4* is due to the large number of variant types that are present across the large *ABCA4* gene and there are no specific mutation “hot spots” that can be targeted.

A significant proportion of STGD1 patients are not found to carry two *ABCA4* variants in *trans.* This will be referred to as missing heritability in this review. Solving this missing heritability is important in understanding the natural history of the disease and its variability in expression, and help identify suitable patients for recruitment to therapeutic trials. The next section will focus on solving this missing heritability and determination of the pathogenicity of variants in *ABCA4*.

### 4.4. Missing Heritability 

Despite the advances in genetic sequencing, genetic testing (see Section 4.1) in *ABCA4* remains difficult and six years ago it was shown that only 65–79% of patients tested are biallelic for the *ABCA4* variants [124,125,126]; 20–25% of patients are monoallelic and in 15% no *ABCA4* variant was identified [127]. This missing heritability could be explained by undetected variants, such as deep intronic variants (Section 4.4.1), structural variants (Section 4.4.2), variants that are incorrectly classified as benign due to their relatively high carrier frequency (hypomorphic alleles) (Section 4.4.3), phenocopies (Section 3), and very rare cases of uniparental isodisomy [97,128,129]. In the following sections, we will discuss and explore the role that previously undetected variants have played towards solving the missing heritability in *ABCA4*. 

#### 4.4.1. Deep Intronic Variants

In an effort to identify the missing variants in *ABCA4*, Braun et al. sequenced RNA obtained from normal human retina to investigate whether variants in sequences near the splice sites of pseudoexons present in very low amounts of retinal mRNA were more susceptible to pathogenic variants. Indeed, this identified five minor splice-site variants (15 alternate exons that each accounted for less than 1% of the total RNA) in the donor retinas, which were all subsequently shown to be pathogenic [130].

Deep intronic variants have since been identified and shown to account for a significant amount of the missing heritability in monoallelic patients. Indeed, deep intronic variants have been identified in approximately 40% of STGD1 patients that were initially found to carry a single *ABCA4* variant [126,130,131], and are more frequently detected in cohorts of monoallelic patients [132]. Moreover, Khan et al. have recently detected deep intronic variants in 15% of their monoallelic patients using single-molecule molecular inversion probes (smMIPs) [97]. SmMIPs are a cost-effective method of detecting genetic variation [95,97].

Currently, the most frequently identified deep intronic variant is c.4253+43G>A [125,131] and has been proposed to be a hypomorphic variant. It has been found to be associated with late-onset disease and a mild phenotype [132,133,134]. The most prevalent deep intronic variant in a study of Belgian patients is the c.4539+2001G>A, and has been found as a second variant in 26% of monoallelic patients [135]. Overall, there are 35 reported deep intronic variants and all but two have been shown to have splice defects in in vitro splice assays [54].

Based on these studies, deep intronic variants will be important in solving the missing heritability in STGD1. However, the highly polymorphic nature of the *ABCA4* gene also means that caution is advised when assigning pathogenicity to deep intronic variants. For example, Zernant et al. found that the c.6006-609T>A variant was not actually pathogenic and was in fact always found in *cis*, with the pathogenic c.4253+43G>A variant as a complex allele [132].

#### 4.4.2. Structural Variants

The first description of a CNV in *ABCA4* was of the c.2654–905_2743+35del (IVS17-905_IVS18+35del) variant reported by Yatsenko et al. [110]. Since then, CNVs and other structural variants have been infrequently reported in *ABCA4* [126,136,137]. Indeed, in one of the most comprehensive studies into SV using smMIP-based sequencing, Khan et al. identified 11 novel CNV in 16 alleles in 448 bi-allelic STGD1 cases (carrying 896 alleles) [97]. This would suggest that structural variants are unlikely to be a significant explanation for missing heritability in STGD1/ABCA4R. Cremers et al. predict that CNVs only account for approximately 1% of pathogenic *ABCA4* variants [54].

#### 4.4.3. Hypomorphic Alleles and Modifiers

As discussed above, *ABCA4* has a high variant carrier frequency, which means that variants with a relatively high allele frequency are typically assigned as benign. It is now thought that some of these variants are hypomorphic alleles and are only pathogenic when certain conditions are met, such as being in *trans* with a severe variant. They are typically associated with a later onset of disease and a milder phenotype [85,127,132]. The c.5603A>T p.(Asn1868Ile) was recently reclassified as hypomorphic despite having a minor allele frequency of 7% in the European population [127] and has been reported in 348 homozygous unaffected individuals [138]. This variant has been found to be the second variant in 40–50% of monoallelic patients [85,127], 80% of monoallelic patients with late-onset disease [127], and has an allele frequency 3–4 times higher in STGD1 patients compared to the general population [85]. The c.5603A>T p.(Asn1868Ile) variant is typically found in *trans* with a severe variant whereby its predicted penetrance is 2.4% [85,139].

The c.5603A>T p.(Asn1868Ile) has been reported to form complex alleles with c.2588G>C p.(Gly863Ala) [85,127,140], c.5461-10T>C [127,141], c.4496G>A [142], c.2564G>A [115], and c.769-784C>T [133]. Zernant et al. proposed that this complex allele has a variable phenotype depending on the severity of the variant in *trans* and that both variants in the complex allele could have a synergistic effect due to both variants being predicted to affect protein folding and exerting their effect on the ABCA4 protein on the same side of the photoreceptor outer segment [127]. It has also been suggested that c.2588G>C p.(Gly863Ala) is a modifier that only causes disease when it is in *cis* with c.5603A>T p.(Asn1868Ile). The c.2588G>C p.(Gly863Ala) variant has also been detected in *cis* in 2/29 alleles with the c.5714+5G>A variant [115,143]. Moreover, the c.5714+5G>A variant was first suggested to have a moderately severe effect [144] and has been observed in mildly affected homozygous STGD1 patients [115].

It is clear that identifying hypomorphic alleles will be an important step towards solving the missing heritability in STGD1. However, deciding whether a variant is truly hypomorphic or a benign polymorphic variant poses a significant interpretation task for a laboratory. Indeed, caution is advised when assigning a variant as hypomorphic in order to avoid incorrect diagnosis. This highlights the importance of developing a pipeline using various bioinformatic algorithms as well as laboratory-based techniques to predict the pathogenicity of variants. These will be outlined in the following section. This approach is underpinned by a need for accurate phenotyping along with family segregation studies. 

## 5. Pathogenicity and Severity of *ABCA4* Variants

*ABCA4* is a large polymorphic gene, meaning that “benign” missense variants are frequently identified. This complicates knowing which variants are truly pathogenic as many *ABCA4* variants are “private” variants [20]. Segregation studies within families are not always able to elucidate the pathogenicity of the identified variant [126]. Genotype–phenotype correlation studies in STGD1 are difficult as patients rarely have the same combination of *ABCA4* variants or are homozygous for the same variant due to the large number of variants seen in *ABCA4* [20,114]. This means that many novel variants get assigned as a variant of unknown significance (VUS) [103]. Indeed, Braun et al. found that 65% of the *ABCA4* variants in their cohort were only identified once [130].

**Figure 5:**
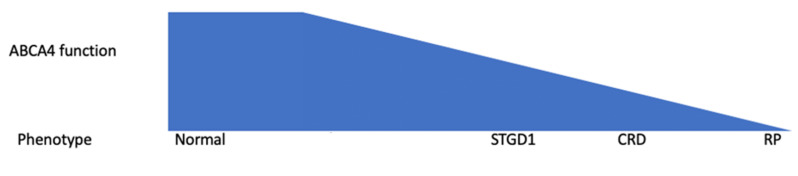
Schematic diagram illustrating the correlation between the amount of functional ABCA4 protein and the phenotype based on early studies that predicted that a lower amount of functioning ABCA4 protein was associated with more severe phenotypes. Figure adapted from Maugeri et al. [53].

Early studies suggested that the phenotypic severity was inversely proportional to the amount of functional ABCA4 protein (see Figure 5). Severe variants were associated with severe phenotypes, such as cone-rod dystrophy [61], retinitis pigmentosa [59,60], choriocapillaris dystrophy [101], and rapid-onset chorioretinopathy [63], whilst missense variants were thought to lead to a milder phenotype because they produce some functioning protein [53,145,146].

However, some missense variants have been reported to cause more severe disease with a significantly earlier age of onset compared to other missense variants [117] and more severe phenotypes, such as cone rod dystrophy and RP [20]. Similarly, the c.768G>T p.(Leu257Valfs*17) variant results in a severe phenotype and has been shown to affect splicing, resulting in a 35 bp exon 6 elongation, despite it being a synonymous change [147]. Deep intronic variants similarly have a variable severity, as some with a partial effect are mild [125,133] whilst those with full effect are null alleles and can be considered as severe variants [125,126].

The age of onset has been used to predict the severity of variants [114,148] as more severe variants are identified in patients with an early age of onset [90,117]. However, this observation may be affected by recall bias. Indeed, some patients with foveal sparing disease may not present despite having atrophic macular lesions [149]. Other methods to predict the severity of variants include predicting the severity based on the amino acid change, using prediction software, and in vitro functional studies.

### 5.1. Predicting Pathogenicity 

A number of techniques are used to assesses variant pathogenicity. Commonly used algorithms include grading based on the American College of Medical Genetics Criteria [150], allele frequency within populations, in silico analysis programmes, conservation of the region across different species, and calculating the combined annotation-dependant duplication (CADD) score. Pathogenic variants are expected to have a low allele frequency within the population; however, this method will miss the hypomorphic alleles, which can have a relatively high frequency in the general population. In silico analysis programmes are used to predict the pathogenicity of variants; however, these programmes can give differing predictions and historically could only correctly predict splicing defects in 70–80% of variants [151]. However, technological advances have meant that predictions of splicing defects have improved with the introduction of newer software, such as spliceAI, which utilises deep-learning techniques [152]. Moreover, using strict screening criteria could mean some causal variants are missed. Genetic regions that are highly conserved across different species are expected to be important regions and variants occurring within these regions are assumed to more likely affect the function of proteins [96]. A CADD score > 10 means that the variant would be predicted to be in the top 10% of most deleterious substitutions in the human genome [153].

Fujinami et al. proposed three genotype severity groups (see Table 3) [155] and this classification was used to classify the genotypes of patients recruited to the ProgStar study, which is the largest natural history study on STGD1 [104,156]. Interestingly, Fujinami et al. found that a higher proportion of patients with childhood-onset disease had more severe variants compared to patients with adult onset [154]. Another severity classification (see Table 4) was proposed by Cornelis et al. following a meta-analysis on all the variants on the LOVD database [148]. 

### 5.2. Functional Analysis of ABCA4 Variants

Functional assessment of the *ABCA4* variants is important to determine whether they affect protein function. In silico analyses are used to predict the effect of variants on the protein; however, these are not always accurate [105]. In vitro analysis can have an important role in deciding whether rare *ABCA4* variants can be classified as pathogenic. 

As *ABCA4* is only highly expressed in the retina, obtaining patient tissue to be used for functional assessment of variants is very difficult, meaning that in vitro assays have to be used [157]. Early in vitro assay studies used lymphoblastoid cells that were limited due to the low expression of *ABCA4* in blood [141,158]. Keratinocytes have since been shown to express *ABCA4* at low levels [130] and more recently reverse transcription polymerase chain reaction (RT-PCR) on photoreceptor progenitor cells derived from induced pluripotent stem cells (iPSC) have been used to assess variants [106,159]. More recently, an in vitro assay to investigate the functional effects of NCSS variants and aberrant splicing utilising a midigene system has been developed [147]. This protocol enables any *ABCA4* NCSS variants identified to be tested in their “natural context” and to determine the functional consequence [147].

The value of in vitro functional assessment of variants is demonstrated by the analysis of the c.5461-10T>C variant that is present in up to 7% of European STGD1 patients, making it the third most common *ABCA4* variant in European patients [25,104,113,137]. This variant is associated with an early onset disease and cone rod dystrophy in homozygous patients [106]. However, the pathogenicity of this variant was debated at the time of its discovery by Maugeri et al. in 1999 [53]. It was suggested that it was in linkage disequilibrium with an unknown pathogenic variant [53,118] because it was not predicted to cause a splicing defect using in silico analysis and splicing defects were not identified in early functional studies that used COS7 and lymphoblastoid cells [118]. However, c.5461-10T>C has now been shown to result in a splicing defect that creates a shortened mRNA with alternate splicing and skipping of exons 39 and exons 39–40, producing a truncated protein, p.(Thr1821Valfs*13) and p.(Thr1821Aspfs*6), respectively [106,141], consistent with it being a severe variant.

In vitro protein assays can also be used to assess both the binding to NrPE in the absence of ATP and the dependant ATPase activity [160]. Curtis et al. recently classified missense variants into three classes based on functional assessment studies of expression of ABCA4, basal ATPase activity, and stimulation by N-Ret-PE (Table 5) [161]. Variants assigned to Class 1 were associated with an early age of onset (≤13 years of age) whilst Class 3 variants were associated with late-onset disease (>40 years of age). The authors proposed that Class 3 encompassed hypomorphic alleles as the c.5603A>T p.(Asn1868Ile) variant was found to cause a small but significant reduction in *ABCA4* expression and ATPase activity [161]. Similarly, Garces et al. classified missense variants affecting the TMDs and found that these also resulted in protein misfolding, decreased substrate binding, and reduced ATPase activity [162]. However, a main limitation of in vitro protein assays is that the detergent solubilisation step can denature variants and result in a severe loss of function, as demonstrated by studies on the mild p.(Gly1961Glu) variant [160].

These studies highlight the important role that in vitro analysis plays in assessing the pathogenicity of *ABCA4* variants. Improvements in the ability to analyse the effects of the variants will be important towards assigning pathogenicity to rare missense variants and hypomorphic alleles, thus aiding in confirming a molecular diagnosis in ABCA4R patients and is of relevance for recruitment to therapeutic approaches, which will be discussed in the next section. 

## 6. Therapies

Currently there are no commercially available treatments for ABCA4R/STGD1. Patients are currently advised to avoid supplements containing vitamin A, due to lipofuscin accumulation being seen in *Abca4* knockout mice that were given vitamin A [163]. Wearing protective, dark-tinted glasses in bright conditions is recommended to reduce short wavelength light reaching the retina, thus reducing the risk of light toxicity [164]. Potential treatments currently being investigated include pharmacological interventions, gene therapy, and stem cell-based therapy approaches (see Table 6). Novel therapies are initially investigated in animal models followed by trials in human subjects. Human trials are divided into four phases: Phase I—to assess the safety of the therapy in a small number of subjects; Phase II—to assess efficacy where patients are randomly placed in treatment and placebo arms; Phase III—similarly assesses efficacy but uses a larger cohort of randomized patients; and Phase IV—monitoring the therapy when it becomes available.

Pharmacological therapies for ABCA4R are mainly based on targeting aspects of the visual cycle in order to reduce the accumulation of lipofuscin deposits. Table 6 details the effect of the compound and trial results if published. The main advantage of these potential therapies is that they can be taken orally, meaning they are less invasive. 

Gene therapy approaches employ viral or non-viral vectors to introduce genetic material into cells in order to either replace an abnormal gene, modify a gene, or silence a dominant gene [165,166,167,168,169,170]. Recently, gene therapy using an adeno-associated virus (AAV) vector has been shown to be effective in the treatment of a different IRD called Leber congenital amaurosis (LCA) caused by *RPE65* variants. It has been licensed for use by the US Food and Drug Administration (FDA) [171] and was approved by NICE in the UK in 2019, with treatment already started on the NHS for this specific disease [172]. However, using this approach in *ABCA4* is complicated as the capacity of the AAV vector is approximately 4.8 kb and the *ABCA4* gene is 6.8 kb [173,174]. Attempts to overcome this limitation have included the use of larger vectors, such as lentiviruses [175,176], a dual AAV approach [173,177,178,179], and the use of nanoparticles [174,180,181,182]. These different approaches will be briefly covered in this review and we direct readers to other review articles that cover gene therapy in more depth [180,183,184].

Lentiviruses are retroviruses that can be used to package *ABCA4* due to their carrying capacity of 8kb [185,186]. The Equine Infectious Anemia Virus (EIAV) lentivirus containing the human *ABCA4* gene has been shown to significantly reduce the accumulation of A2E in *Abca4* KO mice [175] and was found to be safe in rabbit and macaque retinas [176]. The only human gene therapy trial in *ABCA4* was carried out by Oxford Biomedica (SAR422459 NCT 01367444) using the EIAV vector, but the results were not published at the time of this review. 

The dual AAV approach splits the *ABCA4* gene between two AAVs that can combine to form the full length *ABCA4* gene in the host [173,177,178,179]. A study by Dyka et al. showed detectable levels of full-length protein in vitro [177]. The *Abca4* KO mouse treatment resulted in expression of ABCA4 within the photoreceptor outer-segments [173,177], expression of ABCA4 beyond the treated region [173], a significant reduction in bisretinoid levels [173,177], and a reduction in AF signal [173]. However, this approach is limited by the dual vector being less able to transduce photoreceptors compared to single AAV vectors [178,179]. In addition, there is reduced promoter activity of the inverted terminal repeats (ITR), resulting in the production of truncated proteins [178,187]. However, recently Trapani et al. found that the inclusion of the CL1 degron led to a significant reduction in truncated proteins [188]. 

Nanoparticles are synthetic vectors that range in size from 10 to 500 nm [180] and with a capacity of between 5.3 and 20.2 kb [181], which can be used to package the wild-type *ABCA4* gene. A review of nanoparticles and other non-viral vectors is beyond the scope of this review and we direct the readers to a review by Charbel Issa and MacLaren for more detailed information [189]. Treatment in *Abca4* KO mice has been show to result in expression of transgene mRNA, expression of ABCA4 within the outer segments of rod and cone photoreceptors, a significant reduction in lipofuscin levels to levels similar to those in WT mice, a significantly thicker outer nuclear layer similar to that in WT mice, reduction in A2E, improved dark adaptation, improved dark adaptation on electrodiagnostic testing (EDT), delayed progression of disease, and the absence of white spots seen on the fundus of *Abca4* KO mice [174,182].

A newer approach to target deep intronic variants is to use anti-sense oligonucleotides (AON). These are short sequences of RNA that can modulate splicing defects by interfering with mRNA processing [190]. This approach has been shown to be effective in correcting the splicing abnormalities in several IRDs, such as LCA secondary to deep intronic variant c.2991+1655A>G; in *CEP290* [191], which is being currently investigated in a clinical trial [192]; c.7595-2144A>G in *USH2A* [193]; and c.610 + 34G>A in *OPA1* [194]. AON-mediated rescue of pseudoexon defects has similarly been shown in deep intronic *ABCA4* variants using HEK293T cells, patient-derived fibroblasts, and induced pluripotent stem cells (iPSCs) [159,195,196,197]. In *ABCA4*, AON rescue was demonstrated for c.859–540C>G, c.4539+1106C>T, c.4539+2001G>A, c.4539+2028C>T, c.5196+1013A>G and c.5196+1056A>G, c.5196+1137G>A, c.5196+1216C>A, and c.5197–557G>T [131,195,196,197]. However, this is a very bespoke approach, as AONs are variant or pseudoexon specific and their efficacy at correcting splicing defects is reduced by a single nucleotide mismatch [159,196].

Stem cell-based therapies in STGD1 aim to replace the diseased or atrophic RPE cells with RPE made from either human embryonic stem cells (hESCs) or iPSCs. Three Phase I/II trials in humans have all found the procedure to be safe [198,199,200,201]. Schwartz et al. found that 8/10 patients had a significant gain in VA but no significant changes to the visual field, electrodiagnostic test results, or reading speeds were detected, despite patients reporting a median of 8–20 points improvement in their National Eye Institute Visual Function Questionnaire [198]. Another approach by Oner et al. was to use suprachoroidal transplantation of adipose tissue-derived mesenchymal stem cells (ADMSC) in patients with AMD and STGD1. No serious adverse events were reported, and all eight treated patients had significant improvements in VA, visual field, and mfERG results [202].

There are a number of different therapeutic approaches that could potentially become available in the future for ABCA4R, thus highlighting the importance of early identification of patients so that they can benefit when therapies become available and receive treatment at an earlier stage of disease. Detection of at least two pathogenic variants in *trans* will be mandatory in order to ensure patients do not undergo therapies that could cause harm without a likely benefit. 

## 7. Discussion 

STGD1/ABCA4R disease is one of the most common causes of IRD but its true prevalence is unknown. The frequently quoted prevalence of 1/10,000 was estimated by Blacharski et al. in 1988, but was not based on a prevalence study [1]. Recently, the British Ophthalmic Surveillance Unit (BOSU) reported the incidence of STGD1 to range between 0.11 and 0.13 per 100,000 individuals per year [222]. Although, the carrier frequency is reported to be as high as 1 in 20 in different populations [3,4].

Stargardt/ABCA4R is considered to be an autosomal recessive disease [2] but Runhart et al. have recently proposed that STGD1 should be considered a polygenic or multifactorial disease based on a reported female sex bias in 25% of their STGD1 cases that had a combination of a mild and null allele [86]. However, Lee et al. did not find a sex bias in their STGD1 cohort and proposed that STGD1 follows a Mendelian inheritance pattern [87]. The phenotypic appearance in ABCA4R is also highly variable, making identifying and diagnosing patients difficult, and this is explored in more detail in our review of multi-modal imaging in ABCA4R [30]. Indeed, patients usually present in early childhood [1,22,23], but onset in adulthood and late adulthood (>45 years of age), with some cases having an age of onset at 80, is being increasingly recognised [26,27,28,29]. Patients with late-onset disease may be misdiagnosed as geographic atrophy/dry age-related macular degeneration [27], and limited availability of genetic testing may mean that not all cases are identified. Thus, ABCA4R may be more prevalent than current estimates.

This is further complicated by the existence of phenocopies, which must be considered especially when only one variant is identified or in families with a pseudo-dominant inheritance pattern. This is because the IRD could be due to variants in other genes, such as *PRPH2* [70] and *CRX* [72], which are associated with an autosomal dominant inheritance pattern, and have variable penetrance. There may be other clinical features in these other disorders that could help distinguish them [30]. Correctly identifying these patients will be important in order to ensure adequate counselling regarding the prognosis, risk to offspring, and explanation regarding the risk of late-onset disease in unaffected relatives, as well as become highly relevant when therapeutic options become available.

However, this is not always straightforward. The complexity stems from a number of factors related to the *ABCA4* gene, including its large size, highly polymorphic nature, and the large number of variants occurring in both the coding and non-coding regions. Patients are also frequently found to have single or no variants in *ABCA4*. The contribution of undetected variants, such as deep intronic variants, structural variants, hypomorphic alleles (incorrectly classified as “benign”), and disorders caused by variants in another gene (phenocopies) all play a role in solving the missing heritability in ABCA4R.

Advances in genetic sequencing, such as NGS and smMIPS, have enabled the detection of deep intronic variants, which have now been shown to be pathogenic [97,126,130,131] and are currently thought to account for 10% of all pathogenic variants in *ABCA4* [54]. The recent classification of c.5603A>T as a hypomorphic allele has highlighted that hypomorphic alleles can explain a significant amount of monoallelic cases. However, care will be needed when classifying variants as hypomorphic in order to avoid incorrectly diagnosing patients with STGD1. Structural variants [110,126,136,137] and uniparental isodisomy [97,128,129] are rarely found in the *ABCA4* gene, which suggests that these changes do not account for a significant proportion of the missing heritability. 

However, the possibility that the detected variants are part of a complex allele rather than in *trans* must be investigated. Complex alleles are thought to be present in approximately 10% of patients [92], which highlights the importance of segregation studies to confirm that the identified variants are in *trans*. Segregation studies to identify whether detected variants form a complex allele rather than in *trans* are indicated (especially more than one IRD gene variant is present). However, this is not always possible. 

Research into the genotype–phenotype correlation and genetic testing in ABCA4R is of significant importance. It was previously thought that missense variants result in a milder phenotype and that deleterious variants that result in truncated proteins are more severe [53]. However, this has not always been found to be the case [117,223]. The large number of variants complicates genotype–phenotype correlation in *ABCA4* as it is rare for patients to have the same combination of variants or be homozygous unless there is a history of consanguinity.

The majority of variants are missense variants, which are difficult to predict the severity of, meaning that many novel missense variants are classified as VUS. The ProgStar study [104] and Cornelis et al. [148] have both devised methods to classify the severity of different combinations of *ABCA4* variants in patients. However, it is still unknown if these predictions accurately correlate to the phenotypic appearance on multi-modal imaging and on photoreceptor function on electrodiagnostic testing.

Predicting the severity of *ABCA4* variants is also difficult; for example, the c.768G>T variant, originally thought to be a synonymous change, was subsequently found to affect splicing and results in a truncated protein p.(Leu257Valfs*17) [147]. This also highlights the limitation of using the Fujinami genotype prediction model [104], which would have classified this variant as mild. The pathogenicity of known variants such as c.2588G>C, p.(Gly863Ala) has also been revaluated as this variant was initially thought to be mild and only pathogenic when in *trans* with a severe variant, but it is now considered to be a benign variant when it is on its own and is only pathogenic when in *cis* with the c.5603A>T, p.(Asn1868Ile) [115,143]. Indeed, the variable phenotype observed in siblings carrying the same variants supports the presence of modifier genes/variants and environmental factors influencing the phenotype [81,82,83,84]. The further role of *ABCA4* variants or other genetic factors that act as modifiers is of great interest but will be difficult to evaluate.

Currently, our ability to assess the effects of specific *ABCA4* variants is limited due to the lack of a crystal structure of the ABCA4 protein [224]. Predictions of the pathogenicity of variants is mostly based on prediction programs, which are not always accurate [151]. The frequency of variants within the general population may lead to hypomorphic alleles being inadvertently removed from the analysis. Moreover, the majority of studies on *ABCA4* have been regarding patients of European descent, and recent studies have shown that the frequencies of different variants differ between countries [104] and ethnicities [103,112]. Further studies in different populations are required, highlighting the importance of ethnicity-specific genome databases to help ascertain whether a variant is a benign polymorphism or pathogenic. Differences in variants between ethnicities can also influence the phenotype. For example, African Americans have been reported to have milder phenotypes and later-onset disease compared to patients of European descent [112]. This variability will be important to consider when counselling patients regarding prognosis and also when screening for variants in the *ABCA4* gene. Moreover, further research will be needed to accurately characterise the disease within different ethnicities to help define its natural history. This will be important in the design of and recruitment into therapeutic trials.

In vitro functional assays can be used to determine the functional effects of variants and recent advances with the creation of patient-derived iPSCs means that investigations are now more accurate, relevant, and reliable [106,159]. However, caution is still required when interpreting the results of these studies as some variants can be denatured by the detergents, thus affecting the outcomes of the studies [160]; these functional assays should be considered “surrogate” studies, as they are unable to measure the flippase activity. Furthermore, the lower amounts of retina-specific factors in iPSCs means that they will not have the same splicing as that in retinal tissue [97]. Nevertheless, these in vitro studies have a useful role in assessing whether rare variants and VUS are truly pathogenic. Functional studies are important in assigning pathogenicity to missense variants and hypomorphic alleles [161]. This information can be used to determine whether patients carrying these variants are eligible to be recruited to trials. Moreover, it will help identify patients with late-onset disease who are sometimes misdiagnosed with age-related macular degeneration who are at risk of being advised to take supplements containing high doses of vitamin A, which can have detrimental effects in STGD1/ABCA4R.

## 8. Conclusions

In conclusion, STGD1/ABCA4R is one of the most common IRDs. It is associated with central visual loss and in some more global retinal disease. Accurate diagnosis is complicated by the high variability in both the genotype and phenotype in *ABCA4*. Genotyping in *ABCA4* is difficult due to the highly polymorphic nature of the gene, the presence of many “private variants”, deep intronic variants, complex alleles, hypomorphic alleles, and “phenocopies”. An accurate genotype–phenotype correlation is important to understand and document the natural history of the disease, providing valuable information when assessing the efficacy of therapies. It is still unknown whether and to what extent the phenotype is also affected by modifiers and environmental factors; these will also play a role in the response to treatment. NGS-based sequencing enables the detection of variants in phenocopy genes that can mimic the appearance of STGD1/ABCA4R and recent advances in genome sequencing are improving the detection rates of *ABCA4* variants. Developments in functional studies are shedding light on the effect of variants on the ABCA4 protein as well as insights into treatment targets. Therapeutic approaches currently under investigation for ABCA4R include pharmacological, gene, gene-targeted, and stem cell-based therapies. A molecularly confirmed diagnosis of STGD1 is key for a diagnosis and recruitment into appropriate therapeutic trials.

## Figures and Tables

**Table 1:** A summary of the *ABCA4* phenocopies.

Phenocopy	Disease	Inheritance pattern	Associated phenotypes	References
*ELOVL4*	STGD2 and STGD3	Autosomal dominant	Macular pigmentary changes and flecks	[64,67]
*PROM1*	STGD4	Autosomal dominant	Cone-rod dystrophy Macular dystrophy Retinitis pigmentosa Bull’s eye maculopathy (BEM) Flecks	[32,65,68,69]
*PRPH2*	Pattern dystrophy	Autosomal dominant	Pattern dystrophy Fleck like lesions that can be confused with STGD1.	[70,71]
*CRX*	Cone-rod dystrophy	Autosomal dominant	BEM	[72,73]
*BEST1*	Bestrophinopathies	Autosomal recessive Autosomal dominant	Widespread vitelliform flecks in autosomal recessive Adult vitelliform lesion in autosomal dominant	[74,75]
*CDH3*	Macular dystrophy	Autosomal recessive	Juvenile onset macular dystrophy with associated hyptrichosis of scalp hair	[76,77]
Hydroxychloroquine retinopathy	Bull’s eye maculopathy	Drug toxicity	Bull’s eye maculopathy	[58]

**Table 2:** The detection rate of *ABCA4* variants in patients with an STGD1 phenotype in different populations.

Population	*ABCA4* Allele Detection Rate	Reference
Canada (isolated population Newfoundland)	93%	[115]
Chinese	61–84%	[103,116]
Polish	79%	[117]
Danish	77%	[99]
Portuguese	76%	[100]
Spain	76%	[98]
Germany	74%	[118]
Mexico	74%	[119]
USA	50–75%	[120,121]
Hungarian	65.7%	[122]
South Africa	62%	[123]
Canadian	59%	[116]
French Canadian	33%	[116]

**Table 3:** The three genotype severity classifications described by Fujinami et al. and the proportion of patients with each of these severities [104,154].

	Description	Proportion of ProgStar cohort	Proportion in Adult Cohort with Age of Onset >17 Years [154]	Proportion in Paediatric Cohort with Age of Onset <17 years [154]
A	Two or more severe or null variants	5.7%	1.6%	20.6%
B	One severe/null variant and at least one missense or in frame deletion insertion	44.4%	40.6%	44.1%
C	Two or more missense or in frame insertion/deletion variants	49.8%	54.7%	35.2%

**Table 4:** Summary of the classification criteria used by Cornelis at al. to decide the genotype severity [148].

Class	Description	Pathogenicity
1	Truncating variant	Pathogenic
2	Non-truncating but enriched in *ABCA4*-LOVD data set compared to nFE ExAc control group	Likely pathogenic
3	Non-truncating variant that is more frequent in the *ABCA4*-LOVD dataset compared to ExAc control group but not significantly enriched	Uncertain significance
4	Variant had higher frequency in nFE ExAc control group than *ABCA4*-LOVD data set	Likely benign
5	Variant has a frequency > 0.005 in nFE ExAC population and not a known mild *ABCA4* variant	Benign

**Table 5:** A summary of the severity classification of the variants described by Curtis et al. [161].

Class	ABCA4 Expression	Basal ATPase Activity	Stimulation by N-Ret-PE
1	Significantly reduced	<50%	Not stimulated
2	Partial reduction	50–80%	Modestly stimulated
3	Comparable to WT	Comparable to WT	Comparable to WT

**Table 6:** A summary of the current pharmacological therapeutic studies in *ABCA4.*

Therapy	Mechanism of action	Result	Reference
Deuterated vitamin A	Vitamin A is deuterated at the carbon 20 position which strengthens the bond to the retinaldehyde-PE Schiff base which slows the production of A2E production and also provides more time for the Schiff base to be returned to the visual cycle	*Abca4* KO Mice: Decreased A2E Decreased ATR dimer Decreased lipofuscin Decreased autofluorescence signal Improvement in electrodiagnostic testing Phase 1 study in humans Results awaited	[203,204,205]
RBP4 antagonists	Retinol binding protein 4 antagonists inhibit the binding of all-*trans*-retinol to RBP4 in the serum thus decreasing the transport of all-*trans*-retinol to the RPE and as a result decreasing bisretinoid production	Fenretidine in *Abca4* KO: Decreased A2E Decreased AF A1120 in Abca4 KO: Decreased lipofuscin Possibly better antagonist than fenretidine	[206] [207]
Emixustat hydrochloride	Emixustat hydrochloride inhibits the RPE65 protein which reduces 11-*cis*-retinal thus all-*trans*-retinal and the subsequent A2E accumulation	*Abca4* KO mice: Decrease A2E Decreased lipofuscin AF Phase 1 trial “Dose related suppression of rod b wave amplitude recovery post photobleaching”	[208] [209]
4-methylpyrazole	Inhibits alcohol dehydrogenase	Humans No published results on the treated STGD1 patients	
Isotretinoin	Inhibits 11-*cis*-retinol dehydrogenase in the RPE thus decreasing 11-cis-retinaldehyde production and rhodopsin regeneration	In *Abca4* KO Decreased A2E Decreased lipofuscin Delayed dark adaptation Protect photoreceptors from phototoxicity	[210,211]
Saffron	Counteract oxidative damage through the carotenoid constituents crocins and crocetin	Shown to be safe in a double-blind placebo-controlled trial	[212]
VX-809	Increase expression of ABCA4 protein in cells containing the p.(Ala1038Val) and p. (Gly1961Glu) variants	Increased expression of ABCA4 protein in HEK293T cells	[213]
Amine containing drugs	Sequesters all-trans-retinal by reacting with the aldehyde group and forming an inactive Schiff-base and those that compete with PE thus preventing A2E production	*Abca4* KOPreserved retinal architecture in treated mice compared to untreated	[214]
Ticagrelor	Exposure to A2E increases the lysosomal pH which affects degeneration of the photoreceptors. Ticagrlor targets this by inhibiting the P2Y12 receptor to lower the lysosomal pH.	*Abca4* KO Thicker outer nuclear layer Decreased AF Improved a and b wave responses on EDT Reduced lysosomal pH Higher LAMP1 expression (meaning improved lysosomal function)	[215,216]
Soraprazan	Reversible fast acting inhibitor H+, K+ ATPase that was noted to decrease lipofuscin deposits.	Decreased lipofuscin in RPE in treated monkeys and mice	[217,218]
Zimura®	Target C5 complement pathway to prevent formation of the membrane attack complex thus reducing cell death following activation of the complement pathway by A2E and bistretinoids	Results of Phase IIb study awaited	[219,220]
Omega 3 fatty acid supplementation	Omega 3 fatty acids are thought to be important for general retinal function	Trial results awaited	NCT03297515
Docosahexaenoic acid (DHA) supplementation	DHA normally has a high concentration in the retina and is important towards retinal function	A trial showed no improvement in retinal function	[221]

## Data Availability

Data sharing not applicable.

## References

[1] Blacharski P. (1988). Retinal Dystrophies and Degenerations.

[2] Allikmets R., Singh N., Sun H., Shroyer N.F., Hutchinson A., Chidambaram A., Gerrard B., Baird L., Stauffer D., Peiffer A. (1997). A photoreceptor cell-specific ATP-binding transporter gene (ABCR) is mutated in recessive Starqardt macular dystrophy. Nat. Genet..

[3] Yatsenko A.N., Shroyer N.F., Lewis R.A., Lupski J.R. (2001). Late-onset Stargardt disease is associated with missense mutations that map outside known functional regions of ABCR (ABCA4). Hum. Genet..

[4] Jaakson K., Zernant J., Külm M., Hutchinson A., Tonisson N., Glavac D., Ravnik-Glavac M., Hawlina M., Meltzer M.R., Caruso R.C. (2003). Genotyping microarray (gene chip) for the ABCR (ABCA4) gene. Hum. Mutat..

[5] Stargardt K. (1909). Über familiäre, progressive Degeneration in der Maculagegend des Auges. Albrecht Graefes Arch. Ophthalmol..

[6] Franceschetti A., Francois J. (1965). Fundus flavimaculatus. Arch. Ophtalmol. Rev. Gen. Ophtalmol..

[7] Franceschetti A. (1965). A special form of tapetoretinal degeneration: Fundus flavimaculatus. Trans. Am. Acad. Ophthalmol. Otolaryngol..

[8] Kaplan J., Gerber S., Larget-Piet D., Rozet J.-M., Dollfus H., Dufier J.-L., Odent S., Postel-Vinay A., Janin N., Briard M.-L. (1993). A gene for Stargardt’s disease (fundus flavimaculatus) maps to the short arm of chromosome 1. Nat. Genet..

[9] Hadden O.B., Gass J.D. (1976). Fundus flavimaculatus and Stargardt’s disease. Am. J. Ophthalmol..

[10] Noble K.G., Carr R.E. (1979). Stargardt’s disease and fundus flavimaculatus. Arch. Ophthalmol..

[11] Gerber S., Rozet J.M., Bonneau D., Souied E., Camuzat A., Dufier J.L., Amalric P., Weissenbach J., Munnich A., Kaplan J. (1995). A gene for late-onset fundus flavimaculatus with macular dystrophy maps to chromosome 1p13. Am. J. Hum. Genet..

[12] Hoyng C.B., Poppelaars F., Van de Pol T.J.R., Kremer H., Pinckers A.J.L.G., Deutman A.F., Cremers F.P.M. (1996). Genetic fine mapping of the gene for recessive Stargardt disease. Hum. Genet..

[13] Illing M., Molday L.L., Molday R.S. (1997). The 220-kDa rim protein of retinal rod outer segments is a member of the ABC transporter superfamily. J. Biol. Chem..

[14] Sun H., Nathans J. (1997). Stargardt’s ABCR is localized to the disc membrane of retinal rod outer segments. Nat. Genet..

[15] Molday L.L., Rabin A.R., Molday R.S. (2000). ABCR expression in foveal cone photoreceptors and its role in Stargardt macular dystrophy. Nat. Genet..

[16] Lois N., Halfyard A.S., Bird A.C., Holder G.E., Fitzke F.W. (2004). Fundus autofluorescence in Stargardt macular dystrophy-fundus flavimaculatus. Am. J. Ophthalmol..

[17] Cideciyan A.V., Swider M., Aleman T.S., Sumaroka A., Schwartz S.B., Roman M.I., Milam A.H., Bennett J., Stone E.M., Jacobson S.G. (2005). ABCA4-associated retinal degenerations spare structure and function of the human parapapillary retina. Investig. Ophthalmol. Vis. Sci..

[18] Schwoerer J., Secretan M., Zografos L., Piguet B. (2000). Indocyanine green angiography in Fundus flavimaculatus. Ophthalmologica.

[19] Khan K.N., Kasilian M., Mahroo O.A.R., Tanna P., Kalitzeos A., Robson A.G., Tsunoda K., Iwata T., Moore A.T., Fujinami K. (2018). Early Patterns of Macular Degeneration in ABCA4-Associated Retinopathy. Ophthalmology.

[20] Joo K., Seong M.-W., Park K.H., Park S.S., Woo S.J. (2019). Genotypic profile and phenotype correlations of ABCA4-associated retinopathy in Koreans. Mol. Vis..

[21] Al-Ani H.H., Sheck L., Vincent A.L. (2021). Peripheral pigmented lesions in ABCA4-associated retinopathy. Ophthalmic Genet..

[22] Kong X., Strauss R.W., Michaelides M., Cideciyan A.V., Sahel J.-A., Muñoz B., West S., Scholl H.P.N., Wolfson Y., Bittencourt M. (2016). Visual Acuity Loss and Associated Risk Factors in the Retrospective Progression of Stargardt Disease Study (ProgStar Report No. 2). Ophthalmology.

[23] Kong X., Strauss R.W., Cideciyan A.V., Michaelides M., Sahel J.-A., Munoz B., Ahmed M., Ervin A.M., West S.K., Cheetham J.K. (2017). Visual Acuity Change over 12 Months in the Prospective Progression of Atrophy Secondary to Stargardt Disease (ProgStar) Study: ProgStar Report Number 6. Ophthalmology.

[24] Lambertus S., Van Huet R.A., Bax N.M., Hoefsloot L.H., Cremers F.P., Boon C.J., Klevering B.J., Hoyng C.B. (2015). Early-onset stargardt disease: Phenotypic and genotypic characteristics. Ophthalmology.

[25] Utz V.M., Coussa R.G., Marino M.J., Chappelow A.V., Pauer G.J., Hagstrom S.A., Traboulsi E.I. (2014). Predictors of visual acuity and genotype-phenotype correlates in a cohort of patients with Stargardt disease. Br. J. Ophthalmol..

[26] Fujinami K., Sergouniotis P.I., Davidson A.E., Wright G., Chana R.K., Tsunoda K., Tsubota K., Egan C.A., Robson A.G., Moore A.T. (2013). Clinical and molecular analysis of Stargardt disease with preserved foveal structure and function. Am. J. Ophthalmol..

[27] Haaften S.C.W.-V., Boon C.J., Cremers F.P., Hoefsloot L.H., Den Hollander A.I., Hoyng C.B. (2012). Clinical and genetic characteristics of late-onset Stargardt’s disease. Ophthalmology.

[28] Kong X., West S.K., Strauss R.W., Munoz B., Cideciyan A.V., Michaelides M., Ho A., Ahmed M., Schönbach E.M., Cheetham J.K. (2017). Progression of Visual Acuity and Fundus Autofluorescence in Recent-Onset Stargardt Disease: ProgStar Study Report #4. Ophthalmol. Retina.

[29] Testa F., Melillo P., Di Iorio V., Orrico A., Attanasio M., Rossi S., Simonelli F. (2014). Macular function and morphologic features in juvenile stargardt disease: Longitudinal study. Ophthalmology.

[30] Al-khuzaei S., Shah M., Foster C.R., Yu J., Broadgate S., Halford S., Downes S.M. (2021). The role of multimodal imaging and vision function testing in ABCA4 related retinopathies and their relevance to future therapeutic interventions. Ther. Adv. Ophthalmol..

[31] Stone E.M., Nichols B.E., Kimura A.E., Weingeist T.A., Drack A., Sheffield V.C. (1994). Clinical features of a Stargardt-like dominant progressive macular dystrophy with genetic linkage to chromosome 6q. Arch. Ophthalmol..

[32] Kniazeva M., Chiang M.F., Morgan B., Anduze A.L., Zack D.J., Han M., Zhang K. (1999). A new locus for autosomal dominant stargardt-like disease maps to chromosome 4. Am. J. Hum. Genet..

[33] Tsybovsky Y., Molday R.S., Palczewski K. (2010). The ATP-binding cassette transporter ABCA4: Structural and functional properties and role in retinal disease. Adv. Exp. Med. Biol..

[34] Linton K.J. (2007). Structure and function of ABC transporters. Physiology.

[35] Molday R.S., Zhang K. (2010). Defective lipid transport and biosynthesis in recessive and dominant Stargardt macular degeneration. Prog. Lipid Res..

[36] Tsybovsky Y., Orban T., Molday R.S., Taylor D., Palczewski K. (2013). Molecular Organization and ATP-Induced Conformational Changes of ABCA4, the Photoreceptor-Specific ABC Transporter. Structure.

[37] Molday R.S., Hejtmancik J.F., Nickerson J.M. (2015). Chapter Twenty-Four—Insights into the Molecular Properties of ABCA4 and Its Role in the Visual Cycle and Stargardt Disease. Progress in Molecular Biology and Translational Science.

[38] Molday R.S., Zhong M., Quazi F. (2009). The role of the photoreceptor ABC transporter ABCA4 in lipid transport and Stargardt macular degeneration. Biochim. Biophys. Acta.

[39] Rattner A., Smallwood P.M., Nathans J. (2000). Identification and characterization of all-trans-retinol dehydrogenase from photoreceptor outer segments, the visual cycle enzyme that reduces all-trans-retinal to all-trans-retinol. J. Biol. Chem..

[40] McBee J.K., Palczewski K., Baehr W., Pepperberg D.R. (2001). Confronting complexity: The interlink of phototransduction and retinoid metabolism in the vertebrate retina. Prog. Retin. Eye Res..

[41] Saari J.C. (2000). Biochemistry of visual pigment regeneration: The Friedenwald lecture. Investig. Ophthalmol. Vis. Sci..

[42] Poincelot R.P., Millar P.G., Kimbel R.L., Abrahamson E.W. (1969). Lipid to protein chromophore transfer in the photolysis of visual pigments. Nature.

[43] Anderson R.E., Maude M.B. (1970). Phospholipids of bovine outer segments. Biochemistry.

[44] Beharry S., Zhong M., Molday R.S. (2004). N-retinylidene-phosphatidylethanolamine is the preferred retinoid substrate for the photoreceptor-specific ABC transporter ABCA4 (ABCR). J. Biol. Chem..

[45] Quazi F., Lenevich S., Molday R.S. (2012). ABCA4 is an N-retinylidene-phosphatidylethanolamine and phosphatidylethanolamine importer. Nat. Commun..

[46] Pollock N.L., Callaghan R. (2011). The lipid translocase, ABCA4: Seeing is believing. FEBS J..

[47] Ben-Shabat S., Parish C.A., Vollmer H.R., Itagaki Y., Fishkin N., Nakanishi K., Sparrow J.R. (2002). Biosynthetic studies of A2E, a major fluorophore of retinal pigment epithelial lipofuscin. J. Biol. Chem..

[48] Sparrow J.R., Kim S.R., Cuervo A.M., Bandhyopadhyayand U. (2008). A2E, a pigment of RPE lipofuscin, is generated from the precursor, A2PE by a lysosomal enzyme activity. Adv. Exp. Med. Biol..

[49] Mata N.L., Weng J., Travis G.H. (2000). Biosynthesis of a major lipofuscin fluorophore in mice and humans with ABCR-mediated retinal and macular degeneration. Proc. Natl. Acad. Sci. USA.

[50] Sparrow J.R., Boulton M. (2005). RPE lipofuscin and its role in retinal pathobiology. Exp. Eye Res..

[51] Eldred G.E., Lasky M.R. (1993). Retinal age pigments generated by self-assembling lysosomotropic detergents. Nature.

[52] Quazi F., Molday R.S. (2014). ATP-binding cassette transporter ABCA4 and chemical isomerization protect photoreceptor cells from the toxic accumulation of excess 11-cis-retinal. Proc. Natl. Acad. Sci. USA.

[53] Maugeri A., Van Driel M.A., Van de Pol D.J., Klevering B.J., Van Haren F.J., Tijmes N., Bergen A.A., Rohrschneider K., Blankenagel A., Pinckers A.J. (1999). The 2588G→C mutation in the ABCR gene is a mild frequent founder mutation in the Western European population and allows the classification of ABCR mutations in patients with Stargardt disease. Am. J. Hum. Genet..

[54] Cremers F.P.M., Lee W., Collin R.W.J., Allikmets R. (2020). Clinical spectrum, genetic complexity and therapeutic approaches for retinal disease caused by ABCA4 mutations. Prog. Retin. Eye Res..

[55] Khan M., Cremers F.P.M. (2020). ABCA4-Associated Stargardt Disease. Klin. Monbl. Augenheilkd..

[56] Sears A.E., Bernstein P.S., Cideciyan A.V., Hoyng C., Issa P.C., Palczewski K., Rosenfeld P.J., Sadda S., Schraermeyer U., Sparrow J.R. (2017). Towards Treatment of Stargardt Disease: Workshop Organized and Sponsored by the Foundation Fighting Blindness. Transl. Vis. Sci. Technol..

[57] Duncker T., Tsang S.H., Lee W., Zernant J., Allikmets R., Delori F.C., Sparrow J.R. (2015). Quantitative Fundus Autofluorescence Distinguishes ABCA4-Associated and Non–ABCA4-Associated Bull’s-Eye Maculopathy. Ophthalmology.

[58] Noupuu K., Lee W., Zernant J., Greenstein V.C., Tsang S., Allikmets R. (2016). Recessive Stargardt disease phenocopying hydroxychloroquine retinopathy. Graefes Arch. Clin. Exp. Ophthalmol..

[59] Martínez-Mir A., Paloma E., Allikmets R., Ayuso C., Del Rio T., Dean M., Vilageliu L., Gonzàlez-Duarte R., Balcells S. (1998). Retinitis pigmentosa caused by a homozygous mutation in the Stargardt disease gene ABCR. Nat. Genet..

[60] Fukui T., Yamamoto S., Nakano K., Tsujikawa M., Morimura H., Nishida K., Ohguro N., Fujikado T., Irifune M., Kuniyoshi K. (2002). ABCA4 gene mutations in Japanese patients with Stargardt disease and retinitis pigmentosa. Investig. Ophthalmol. Vis. Sci..

[61] Simonelli F., Testa F., Zernant J., Nesti A., Rossi S., Rinaldi E., Allikmets R. (2004). Association of a homozygous nonsense mutation in the ABCA4 (ABCR) gene with cone-rod dystrophy phenotype in an Italian family. Ophthalmic Res..

[62] Lee W., Zernant J., Nagasaki T., Tsang S.H., Allikmets R. (2018). Deep Scleral Exposure: A Degenerative Outcome of End-Stage Stargardt Disease. Am. J. Ophthalmol..

[63] Tanaka K., Lee W., Zernant J., Schuerch K., Ciccone L., Tsang S.H., Sparrow J.R., Allikmets R. (2018). The Rapid-Onset Chorioretinopathy Phenotype of ABCA4 Disease. Ophthalmology.

[64] Zhang K., Kniazeva M., Han M., Li W., Yu Z., Yang Z., Li Y., Metzker M.L., Allikmets R., Zack D.J. (2001). A 5-bp deletion in ELOVL4 is associated with two related forms of autosomal dominant macular dystrophy. Nat. Genet..

[65] Yang Z., Chen Y., Lillo C., Chien J., Yu Z., Michaelides M., Klein M., Howes K.A., Li Y., Kaminoh Y. (2008). Mutant prominin 1 found in patients with macular degeneration disrupts photoreceptor disk morphogenesis in mice. J. Clin. Investig..

[66] Zhang K., Bither P.P., Park R., Donoso L.A., Seidman J.G., Seidman C.E. (1994). A dominant Stargardt’s macular dystrophy locus maps to chromosome 13q34. Arch. Ophthalmol..

[67] Maugeri A., Meire F., Hoyng C.B., Vink C., Van Regemorter N., Karan G., Yang Z., Cremers F.P.M., Zhang K. (2004). A Novel Mutation in the ELOVL4 Gene Causes Autosomal Dominant Stargardt-like Macular Dystrophy. Investig. Ophthalmol. Vis. Sci..

[68] Del Pozo-Valero M., Martin-Merida I., Jimenez-Rolando B., Arteche A., Avila-Fernandez A., Blanco-Kelly F., Riveiro-Alvarez R., Van Cauwenbergh C., De Baere E., Rivolta C. (2019). Expanded Phenotypic Spectrum of Retinopathies Associated with Autosomal Recessive and Dominant Mutations in PROM1. Am. J. Ophthalmol..

[69] Michaelides M., Johnson S., Poulson A., Bradshaw K., Bellmann C., Hunt D.M., Moore A.T. (2003). An autosomal dominant bull’s-eye macular dystrophy (MCDR2) that maps to the short arm of chromosome 4. Investig. Ophthalmol. Vis. Sci..

[70] Boon C.J., Van Schooneveld M.J., Den Hollander A.I., Van Lith-Verhoeven J.J., Zonneveld-Vrieling M.N., Theelen T., Cremers F.P., Hoyng C.B., Klevering B.J. (2007). Mutations in the peripherin/RDS gene are an important cause of multifocal pattern dystrophy simulating STGD1/fundus flavimaculatus. Br. J. Ophthalmol..

[71] Ibanez Iv M.B., De Guimarães T.A.C., Capasso J., Bello N., Levin A.V. (2020). Stargardt misdiagnosis: How ocular genetics helps. Am. J. Med. Genet. Part A.

[72] Al-Khuzaei S., Hudspith K.A.Z., Broadgate S., Shanks M.E., Clouston P., Németh A.H., Halford S., Downes S.M. (2021). Targeted next generation sequencing and family survey enable correct genetic diagnosis in CRX associated macular dystrophy—A case report. BMC Ophthalmol..

[73] Wolock C.J., Stong N., Ma C.J., Nagasaki T., Lee W., Tsang S.H., Kamalakaran S., Goldstein D.B., Allikmets R. (2019). A case-control collapsing analysis identifies retinal dystrophy genes associated with ophthalmic disease in patients with no pathogenic ABCA4 variants. Genet. Med..

[74] Shah M., Broadgate S., Shanks M., Clouston P., Yu J., MacLaren R.E., Németh A.H., Halford S., Downes S.M. (2020). Association of Clinical and Genetic Heterogeneity With BEST1 Sequence Variations. JAMA Ophthalmol..

[75] Rahman N., Georgiou M., Khan K.N., Michaelides M. (2019). Macular dystrophies: Clinical and imaging features, molecular genetics and therapeutic options. Br. J. Ophthalmol..

[76] Hull S., Arno G., Robson A.G., Broadgate S., Plagnol V., McKibbin M., Halford S., Michaelides M., Holder G.E., Moore A.T. (2016). Characterization of CDH3-Related Congenital Hypotrichosis With Juvenile Macular Dystrophy. JAMA Ophthalmol..

[77] Halford S., Holt R., Németh A.H., Downes S.M. (2012). Homozygous Deletion in CDH3 and Hypotrichosis With Juvenile Macular Dystrophy. Arch. Ophthalmol..

[78] Duncker T., Tsang S.H., Woods R.L., Lee W., Zernant J., Allikmets R., Delori F.C., Sparrow J.R. (2015). Quantitative Fundus Autofluorescence and Optical Coherence Tomography in PRPH2/RDS- and ABCA4-Associated Disease Exhibiting Phenotypic Overlap. Investig. Ophthalmol. Vis. Sci..

[79] Yusuf I.H., Sharma S., Luqmani R., Downes S.M. (2017). Hydroxychloroquine retinopathy. Eye.

[80] Mittra R., Mieler W. (2012). Drug Toxicity of the Posterior Segment. Retina.

[81] Tracewska A.M., Kocyła-Karczmarewicz B., Rafalska A., Murawska J., Jakubaszko-Jablonska J., Rydzanicz M., Stawiński P., Ciara E., Khan M.I., Henkes A. (2019). Genetic Spectrum of ABCA4-Associated Retinal Degeneration in Poland. Genes.

[82] Sodi A., Bini A., Passerini I., Puccioni M., Torricelli F., Menchini U. (2008). Variable expressivity of abca4 gene mutations in an italian family with stargardt disease. Retin. Cases Brief Rep..

[83] Valkenburg D., Runhart E.H., Bax N.M., Liefers B., Lambertus S.L., Sanchez C.I., Cremers F.P.M., Hoyng C.B. (2019). Highly Variable Disease Courses in Siblings with Stargardt Disease. Ophthalmology.

[84] Passerini I., Sodi A., Giambene B., Mariottini A., Menchini U., Torricelli F. (2010). Novel mutations in of the ABCR gene in italian patients with Stargardt disease. Eye.

[85] Runhart E.H., Sangermano R., Cornelis S.S., Verheij J.B.G.M., Plomp A.S., Boon C.J.F., Lugtenberg D., Roosing S., Bax N.M., Blokland E.A.W. (2018). The Common ABCA4 Variant p.Asn1868Ile Shows Nonpenetrance and Variable Expression of Stargardt Disease When Present in trans With Severe Variants. Investig. Ophthalmol. Vis. Sci..

[86] Runhart E.H., Khan M., Cornelis S.S., Roosing S., Del Pozo-Valero M., Lamey T.M., Liskova P., Roberts L., Stöhr H., Klaver C.C.W. (2020). Association of Sex With Frequent and Mild ABCA4 Alleles in Stargardt Disease. JAMA Ophthalmol..

[87] Lee W., Zernant J., Nagasaki T., Allikmets R. (2021). Reevaluating the Association of Sex With ABCA4 Alleles in Patients With Stargardt Disease. JAMA Ophthalmol..

[88] Gerber S., Rozet J.M., Van de Pol T.J., Hoyng C.B., Munnich A., Blankenagel A., Kaplan J., Cremers F.P. (1998). Complete exon-intron structure of the retina-specific ATP binding transporter gene (ABCR) allows the identification of novel mutations underlying Stargardt disease. Genomics.

[89] Ernest P.J.G., Boon C.J.F., Klevering B.J., Hoefsloot L.H., Hoyng C.B. (2009). Outcome of ABCA4 microarray screening in routine clinical practice. Mol. Vis..

[90] Aguirre-Lamban J., Riveiro-Alvarez R., Maia-Lopes S., Cantalapiedra D., Vallespin E., Avila-Fernandez A., Villaverde-Montero C., Trujillo-Tiebas M.J., Ramos C., Ayuso C. (2009). Molecular analysis of the ABCA4 gene for reliable detection of allelic variations in Spanish patients: Identification of 21 novel variants. Br. J. Ophthalmol..

[91] Duno M., Schwartz M., Larsen P.L., Rosenberg T. (2012). Phenotypic and genetic spectrum of Danish patients with ABCA4-related retinopathy. Ophthalmic Genet..

[92] Shroyer N.F., Lewis R.A., Yatsenko A.N., Wensel T.G., Lupski J.R. (2001). Cosegregation and functional analysis of mutant ABCR (ABCA4) alleles in families that manifest both Stargardt disease and age-related macular degeneration. Hum. Mol. Genet..

[93] Strom S.P., Gao Y.-Q., Martinez A., Ortube C., Chen Z., Nelson S.F., Nusinowitz S., Farber D.B., Gorin M.B. (2012). Molecular diagnosis of putative Stargardt disease probands by exome sequencing. BMC Med. Genet..

[94] Sung Y., Choi S.W., Shim S.H., Song W.K. (2019). Clinical and Genetic Characteristics Analysis of Korean Patients with Stargardt Disease Using Targeted Exome Sequencing. Ophthalmologica.

[95] Khan M., Cornelis S.S., Khan M.I., Elmelik D., Manders E., Bakker S., Derks R., Neveling K., Van de Vorst M., Gilissen C. (2019). Cost-effective molecular inversion probe-based ABCA4 sequencing reveals deep-intronic variants in Stargardt disease. Hum. Mutat..

[96] Broadgate S., Yu J., Downes S.M., Halford S. (2017). Unravelling the genetics of inherited retinal dystrophies: Past, present and future. Prog. Retin. Eye Res..

[97] Khan M., Cornelis S.S., Pozo-Valero M.D., Whelan L., Runhart E.H., Mishra K., Bults F., AlSwaiti Y., AlTalbishi A., De Baere E. (2020). Resolving the dark matter of ABCA4 for 1054 Stargardt disease probands through integrated genomics and transcriptomics. Genet. Med..

[98] Valverde D., Riveiro-Alvarez R., Bernal S., Jaakson K., Baiget M., Navarro R., Ayuso C. (2006). Microarray-based mutation analysis of the ABCA4 gene in Spanish patients with Stargardt disease: Evidence of a prevalent mutated allele. Mol. Vis..

[99] Rosenberg T., Klie F., Garred P., Schwartz M. (2007). N965S is a common ABCA4 variant in Stargardt-related retinopathies in the Danish population. Mol. Vis..

[100] Maia-Lopes S., Aguirre-Lamban J., Castelo-Branco M., Riveiro-Alvarez R., Ayuso C., Silva E.D. (2009). ABCA4 mutations in Portuguese Stargardt patients: Identification of new mutations and their phenotypic analysis. Mol. Vis..

[101] Bertelsen M., Zernant J., Larsen M., Duno M., Allikmets R., Rosenberg T. (2014). Generalized choriocapillaris dystrophy, a distinct phenotype in the spectrum of ABCA4-associated retinopathies. Investig. Ophthalmol. Vis. Sci..

[102] Schulz H.L., Grassmann F., Kellner U., Spital G., Rüther K., Jägle H., Hufendiek K., Rating P., Huchzermeyer C., Baier M.J. (2017). Mutation Spectrum of the ABCA4 Gene in 335 Stargardt Disease Patients From a Multicenter German Cohort—Impact of Selected Deep Intronic Variants and Common SNPs. Investig. Ophthalmol. Vis. Sci..

[103] Hu F.-Y., Li J.-K., Gao F.-J., Qi Y.-H., Xu P., Zhang Y.-J., Wang D.-D., Wang L.-S., Li W., Wang M. (2019). ABCA4 Gene Screening in a Chinese Cohort With Stargardt Disease: Identification of 37 Novel Variants. Front. Genet..

[104] Fujinami K., Strauss R.W., Chiang J.P., Audo I.S., Bernstein P.S., Birch D.G., Bomotti S.M., Cideciyan A.V., Ervin A.M., Marino M.J. (2019). Detailed genetic characteristics of an international large cohort of patients with Stargardt disease: ProgStar study report 8. Br. J. Ophthalmol..

[105] Jonsson F., Westin I.M., Österman L., Sandgren O., Burstedt M., Holmberg M., Golovleva I. (2018). ATP-binding cassette subfamily A, member 4 intronic variants c.4773+3A>G and c.5461-10T>C cause Stargardt disease due to defective splicing. Acta Ophthalmol..

[106] Sangermano R., Bax N.M., Bauwens M., Van den Born L.I., De Baere E., Garanto A., Collin R.W., Goercharn-Ramlal A.S., Dijk A.H.D.E.-V., Rohrschneider K. (2016). Photoreceptor Progenitor mRNA Analysis Reveals Exon Skipping Resulting from the ABCA4 c.5461-10T→C Mutation in Stargardt Disease. Ophthalmology.

[107] Schindler E.I., Nylen E.L., Ko A.C., Affatigato L.M., Heggen A.C., Wang K., Sheffield V.C., Stone E.M. (2010). Deducing the pathogenic contribution of recessive ABCA4 alleles in an outbred population. Hum. Mol. Genet..

[108] Fujinami K., Sergouniotis P.I., Davidson A.E., Mackay D.S., Tsunoda K., Tsubota K., Robson A.G., Holder G.E., Moore A.T., Michaelides M. (2013). The clinical effect of homozygous ABCA4 alleles in 18 patients. Ophthalmology.

[109] Escaramís G., Docampo E., Rabionet R. (2015). A decade of structural variants: Description, history and methods to detect structural variation. Brief. Funct. Genom..

[110] Yatsenko A.N., Shroyer N.F., Lewis R.A., Lupski J.R. (2003). An ABCA4 genomic deletion in patients with Stargardt disease. Hum. Mutat..

[111] Sibley C.R., Blazquez L., Ule J. (2016). Lessons from non-canonical splicing. Nat. Rev. Genet..

[112] Zernant J., Collison F.T., Lee W., Fishman G.A., Noupuu K., Yuan B., Cai C., Lupski J.R., Yannuzzi L.A., Tsang S.H. (2014). Genetic and clinical analysis of ABCA4-associated disease in African American patients. Hum. Mutat..

[113] Roberts L.J., Nossek C.A., Greenberg L.J., Ramesar R.S. (2012). Stargardt macular dystrophy: Common ABCA4 mutations in South Africa--establishment of a rapid genetic test and relating risk to patients. Mol. Vis..

[114] Heathfield L., Lacerda M., Nossek C., Roberts L., Ramesar R.S. (2013). Stargardt disease: Towards developing a model to predict phenotype. Eur. J. Hum. Genet..

[115] Green J.S., O’Rielly D.D., Pater J.A., Houston J., Rajabi H., Galutira D., Benteau T., Sheaves A., Abdelfatah N., Bautista D. (2020). The genetic architecture of Stargardt macular dystrophy (STGD1): A longitudinal 40-year study in a genetic isolate. Eur J. Hum. Genet..

[116] Zaneveld J., Siddiqui S., Li H., Wang X., Wang H., Wang K., Li H., Ren H., Lopez I., Dorfman A. (2015). Comprehensive analysis of patients with Stargardt macular dystrophy reveals new genotype–phenotype correlations and unexpected diagnostic revisions. Genet. Med..

[117] Ścieżyńska A., Oziębło D., Ambroziak A.M., Korwin M., Szulborski K., Krawczyński M., Stawiński P., Szaflik J., Szaflik J.P., Płoski R. (2016). Next-generation sequencing of ABCA4: High frequency of complex alleles and novel mutations in patients with retinal dystrophies from Central Europe. Exp. Eye Res..

[118] Rivera A., White K., Stohr H., Steiner K., Hemmrich N., Grimm T., Jurklies B., Lorenz B., Scholl H.P., Apfelstedt-Sylla E. (2000). A comprehensive survey of sequence variation in the ABCA4 (ABCR) gene in Stargardt disease and age-related macular degeneration. Am. J. Hum. Genet..

[119] Chacón-Camacho O.F., Granillo-Alvarez M., Ayala-Ramírez R., Zenteno J.C. (2013). ABCA4 mutational spectrum in Mexican patients with Stargardt disease: Identification of 12 novel mutations and evidence of a founder effect for the common p.A1773V mutation. Exp. Eye Res..

[120] Lewis R.A., Shroyer N.F., Singh N., Allikmets R., Hutchinson A., Li Y., Lupski J.R., Leppert M., Dean M. (1999). Genotype/phenotype analysis of a photoreceptor-specific ATP-binding cassette transporter gene, ABCR, in Stargardt disease. Am. J. Hum. Genet..

[121] Briggs C.E., Rucinski D., Rosenfeld P.J., Hirose T., Berson E.L., Dryja T.P. (2001). Mutations in ABCR (ABCA4) in patients with Stargardt macular degeneration or cone-rod degeneration. Investig. Ophthalmol. Vis. Sci..

[122] Hargitai J., Zernant J., Somfai G.M., Vamos R., Farkas A., Salacz G., Allikmets R. (2005). Correlation of clinical and genetic findings in Hungarian patients with Stargardt disease. Investig. Ophthalmol. Vis. Sci..

[123] September A.V., Vorster A.A., Ramesar R.S., Greenberg L.J. (2004). Mutation spectrum and founder chromosomes for the ABCA4 gene in South African patients with Stargardt disease. Investig. Ophthalmol. Vis. Sci..

[124] Downes S.M., Packham E., Cranston T., Clouston P., Seller A., Németh A.H. (2012). Detection rate of pathogenic mutations in ABCA4 using direct sequencing: Clinical and research implications. Arch. Ophthalmol..

[125] Nassisi M., Mohand-Saïd S., Andrieu C., Antonio A., Condroyer C., Méjécase C., Varin J., Wohlschlegel J., Dhaenens C.-M., Sahel J.-A. (2019). Prevalence of ABCA4 Deep-Intronic Variants and Related Phenotype in An Unsolved “One-Hit” Cohort with Stargardt Disease. Int. J. Mol. Sci..

[126] Zernant J., Xie Y.A., Ayuso C., Riveiro-Alvarez R., Lopez-Martinez M.-A., Simonelli F., Testa F., Gorin M.B., Strom S.P., Bertelsen M. (2014). Analysis of the ABCA4 genomic locus in Stargardt disease. Hum. Mol. Genet..

[127] Zernant J., Lee W., Collison F.T., Fishman G.A., Sergeev Y.V., Schuerch K., Sparrow J.R., Tsang S.H., Allikmets R. (2017). Frequent hypomorphic alleles account for a significant fraction of ABCA4 disease and distinguish it from age-related macular degeneration. J. Med. Genet..

[128] Fingert J.H., Eliason D.A., Phillips N.C., Lotery A.J., Sheffield V.C., Stone E.M. (2006). Case of Stargardt disease caused by uniparental isodisomy. Arch. Ophthalmol..

[129] Riveiro-Alvarez R., Valverde D., Lorda-Sanchez I., Trujillo-Tiebas M.J., Cantalapiedra D., Vallespin E., Aguirre-Lamban J., Ramos C., Ayuso C. (2007). Partial paternal uniparental disomy (UPD) of chromosome 1 in a patient with Stargardt disease. Mol. Vis..

[130] Braun T.A., Mullins R.F., Wagner A.H., Andorf J.L., Johnston R.M., Bakall B.B., Deluca A.P., Fishman G.A., Lam B.L., Weleber R.G. (2013). Non-exomic and synonymous variants in ABCA4 are an important cause of Stargardt disease. Hum. Mol. Genet..

[131] Sangermano R., Garanto A., Khan M., Runhart E.H., Bauwens M., Bax N.M., Van den Born L.I., Khan M.I., Cornelis S.S., Verheij J.B.G.M. (2019). Deep-intronic ABCA4 variants explain missing heritability in Stargardt disease and allow correction of splice defects by antisense oligonucleotides. Genet. Med..

[132] Zernant J., Lee W., Nagasaki T., Collison F.T., Fishman G.A., Bertelsen M., Rosenberg T., Gouras P., Tsang S.H., Allikmets R. (2018). Extremely hypomorphic and severe deep intronic variants in the ABCA4 locus result in varying Stargardt disease phenotypes. Cold Spring Harb. Mol. Case Stud..

[133] Runhart E.H., Valkenburg D., Cornelis S.S., Khan M., Sangermano R., Albert S., Bax N.M., Astuti G.D.N., Gilissen C., Pott J.-W.R. (2019). Late-Onset Stargardt Disease Due to Mild, Deep-Intronic ABCA4 Alleles. Investig. Ophthalmol. Vis. Sci..

[134] Holtan J.P., Aukrust I., Jansson R.W., Berland S., Bruland O., Gjerde B.L., Stokowy T., Bojovic O., Forsaa V., Austeng D. (2020). Clinical features and molecular genetics of patients with ABCA4-retinal dystrophies. Acta Ophthalmol..

[135] Bauwens M., De Zaeytijd J., Weisschuh N., Kohl S., Meire F., Dahan K., Depasse F., De Jaegere S., De Ravel T., De Rademaeker M. (2015). An Augmented ABCA4 Screen Targeting Noncoding Regions Reveals a Deep Intronic Founder Variant in Belgian Stargardt Patients. Hum. Mutat..

[136] Stenirri S., Battistella S., Fermo I., Manitto M.P., Martina E., Brancato R., Ferrari M., Cremonesi L. (2006). De novo deletion removes a conserved motif in the C-terminus of ABCA4 and results in cone-rod dystrophy. Clin. Chem. Lab. Med..

[137] Zernant J., Schubert C., Im K.M., Burke T., Brown C.M., Fishman G.A., Tsang S.H., Gouras P., Dean M., Allikmets R. (2011). Analysis of the ABCA4 gene by next-generation sequencing. Investig. Ophthalmol. Vis. Sci..

[138] Lek M., Karczewski K.J., Minikel E.V., Samocha K.E., Banks E., Fennell T., O’Donnell-Luria A.H., Ware J.S., Hill A.J., Cummings B.B. (2016). Analysis of protein-coding genetic variation in 60,706 humans. Nature.

[139] Cremers F.P.M., Cornelis S.S., Runhart E.H., Astuti G.D.N. (2018). Author Response: Penetrance of the ABCA4 p.Asn1868Ile Allele in Stargardt Disease. Investig. Ophthalmol. Vis. Sci..

[140] Maugeri A., Flothmann K., Hemmrich N., Ingvast S., Jorge P., Paloma E., Patel R., Rozet J.M., Tammur J., Testa F. (2002). The ABCA4 2588G>C Stargardt mutation: Single origin and increasing frequency from South-West to North-East Europe. Eur. J. Hum. Genet..

[141] Aukrust I., Jansson R.W., Bredrup C., Rusaas H.E., Berland S., Jørgensen A., Haug M.G., Rødahl E., Houge G., Knappskog P.M. (2017). The intronic ABCA4 c.5461-10T>C variant, frequently seen in patients with Stargardt disease, causes splice defects and reduced ABCA4 protein level. Acta Ophthalmol..

[142] Midgley N., Roberts L., Rebello G., Ramesar R. (2020). The impact of the c.5603A>T hypomorphic variant on founder mutation screening of ABCA4 for Stargardt disease in South Africa. Mol. Vis..

[143] Thompson J.A., Chiang J.P., De Roach J.N., McLaren T.L., Chen F.K., Hoffmann L., Campbell I., Lamey T.M. (2019). Analysis of the ABCA4 c.[2588G>C;5603A>T] Allele in the Australian Population. Adv. Exp. Med. Biol.

[144] Cremers F.P., Van de Pol D.J., Van Driel M., Den Hollander A.I., Van Haren F.J., Knoers N.V., Tijmes N., Bergen A.A., Rohrschneider K., Blankenagel A. (1998). Autosomal recessive retinitis pigmentosa and cone-rod dystrophy caused by splice site mutations in the Stargardt’s disease gene ABCR. Hum. Mol. Genet..

[145] Van Driel M.A., Maugeri A., Klevering B.J., Hoyng C.B., Cremers F.P. (1998). ABCR unites what ophthalmologists divide(s). Ophthalmic Genet..

[146] Shroyer N.F., Lewis R.A., Allikmets R., Singh N., Dean M., Leppert M., Lupski J.R. (1999). The rod photoreceptor ATP-binding cassette transporter gene, ABCR, and retinal disease: From monogenic to multifactorial. Vis. Res..

[147] Sangermano R., Khan M., Cornelis S.S., Richelle V., Albert S., Garanto A., Elmelik D., Qamar R., Lugtenberg D., Van den Born L.I. (2018). ABCA4 midigenes reveal the full splice spectrum of all reported noncanonical splice site variants in Stargardt disease. Genome Res..

[148] Cornelis S.S., Bax N.M., Zernant J., Allikmets R., Fritsche L.G., Den Dunnen J.T., Ajmal M., Hoyng C.B., Cremers F.P. (2017). In Silico Functional Meta-Analysis of 5,962 ABCA4 Variants in 3,928 Retinal Dystrophy Cases. Hum. Mutat..

[149] Oldani M., Marchi S., Giani A., Cecchin S., Rigoni E., Persi A., Podavini D., Guerrini A., Nervegna A., Staurenghi G. (2012). Clinical and molecular genetic study of 12 Italian families with autosomal recessive Stargardt disease. Genet. Mol. Res..

[150] Richards S., Aziz N., Bale S., Bick D., Das S., Gastier-Foster J., Grody W.W., Hegde M., Lyon E., Spector E. (2015). Standards and guidelines for the interpretation of sequence variants: A joint consensus recommendation of the American College of Medical Genetics and Genomics and the Association for Molecular Pathology. Genet. Med..

[151] Liu Y.H., Li C.G., Zhou S.F. (2009). Prediction of deleterious functional effects of non-synonymous single nucleotide polymorphisms in human nuclear receptor genes using a bioinformatics approach. Drug Metab. Lett..

[152] Jaganathan K., Panagiotopoulou S.K., McRae J.F., Darbandi S.F., Knowles D., Li Y.I., Kosmicki J.A., Arbelaez J., Cui W., Schwartz G.B. (2019). Predicting Splicing from Primary Sequence with Deep Learning. Cell.

[153] Kircher M., Witten D.M., Jain P., O’Roak B.J., Cooper G.M., Shendure J. (2014). A general framework for estimating the relative pathogenicity of human genetic variants. Nat. Genet..

[154] Fujinami K., Zernant J., Chana R.K., Wright G.A., Tsunoda K., Ozawa Y., Tsubota K., Robson A.G., Holder G.E., Allikmets R. (2015). Clinical and molecular characteristics of childhood-onset Stargardt disease. Ophthalmology.

[155] Fujinami K., Lois N., Mukherjee R., McBain V.A., Tsunoda K., Tsubota K., Stone E.M., Fitzke F.W., Bunce C., Moore A.T. (2013). A longitudinal study of Stargardt disease: Quantitative assessment of fundus autofluorescence, progression, and genotype correlations. Investig. Ophthalmol. Vis. Sci..

[156] Strauss R.W., Ho A., Munoz B., Cideciyan A.V., Sahel J.A., Sunness J.S., Birch D.G., Bernstein P.S., Michaelides M., Traboulsi E.I. (2016). The Natural History of the Progression of Atrophy Secondary to Stargardt Disease (ProgStar) Studies: Design and Baseline Characteristics: ProgStar Report No. 1. Ophthalmology.

[157] Azarian S.M., Travis G.H. (1997). The photoreceptor rim protein is an ABC transporter encoded by the gene for recessive Stargardt’s disease (ABCR). FEBS Lett..

[158] Jonsson F., Burstedt M.S., Sandgren O., Norberg A., Golovleva I. (2013). Novel mutations in CRB1 and ABCA4 genes cause Leber congenital amaurosis and Stargardt disease in a Swedish family. Eur. J. Hum. Genet..

[159] Albert S., Garanto A., Sangermano R., Khan M., Bax N.M., Hoyng C.B., Zernant J., Lee W., Allikmets R., Collin R.W.J. (2018). Identification and Rescue of Splice Defects Caused by Two Neighboring Deep-Intronic ABCA4 Mutations Underlying Stargardt Disease. Am. J. Hum. Genet..

[160] Garces F., Jiang K., Molday L.L., Stöhr H., Weber B.H., Lyons C.J., Maberley D., Molday R.S. (2018). Correlating the Expression and Functional Activity of ABCA4 Disease Variants With the Phenotype of Patients With Stargardt Disease. Investig. Ophthalmol. Vis. Sci..

[161] Curtis S.B., Molday L.L., Garces F.A., Molday R.S. (2020). Functional analysis and classification of homozygous and hypomorphic ABCA4 variants associated with Stargardt macular degeneration. Hum. Mutat..

[162] Garces F.A., Scortecci J.F., Molday R.S. (2020). Functional Characterization of ABCA4 Missense Variants Linked to Stargardt Macular Degeneration. Int. J. Mol. Sci..

[163] Radu R.A., Yuan Q., Hu J., Peng J.H., Lloyd M., Nusinowitz S., Bok D., Travis G.H. (2008). Accelerated accumulation of lipofuscin pigments in the RPE of a mouse model for ABCA4-mediated retinal dystrophies following Vitamin A supplementation. Investig. Ophthalmol. Vis. Sci..

[164] Teussink M.M., Lee M.D., Smith R.T., Van Huet R.A.C., Klaver C.C., Klevering B.J., Theelen T., Hoyng C.B. (2015). The Effect of Light Deprivation in Patients with Stargardt Disease. Am. J. Ophthalmol..

[165] Lee J.H., Wang J.H., Chen J., Li F., Edwards T.L., Hewitt A.W., Liu G.S. (2019). Gene therapy for visual loss: Opportunities and concerns. Prog. Retin. Eye Res..

[166] Campa C., Gallenga C.E., Bolletta E., Perri P. (2017). The Role of Gene Therapy in the Treatment of Retinal Diseases: A Review. Curr. Gene Ther..

[167] Den Hollander A.I., Roepman R., Koenekoop R.K., Cremers F.P.M. (2008). Leber congenital amaurosis: Genes, proteins and disease mechanisms. Prog. Retin. Eye Res..

[168] Sundaram V., Moore A.T., Ali R.R., Bainbridge J.W. (2012). Educational paper. Eur. J. Pediatrics.

[169] Oner A. (2017). Recent Advancements in Gene Therapy for Hereditary Retinal Dystrophies. Turk. J. Ophthalmol..

[170] Kumaran N., Michaelides M., Smith A.J., Ali R.R., Bainbridge J.W.B. (2018). Retinal gene therapy. Br. Med. Bull..

[171] U.S Food and Drug Administration. https://www.fda.gov/news-events/press-announcements/fda-approves-novel-gene-therapy-treat-patients-rare-form-inherited-vision-loss.

[172] NICE NICE Recommends Novel Gene Therapy Treatment for Rare Inherited Eye Disorder. https://www.nice.org.uk/news/article/nice-recommends-novel-gene-therapy-treatment-for-rare-inherited-eye-disorder.

[173] McClements M.E., Barnard A.R., Singh M.S., Issa P.C., Jiang Z., Radu R.A., MacLaren R.E. (2019). An AAV Dual Vector Strategy Ameliorates the Stargardt Phenotype in Adult Abca4(-/-) Mice. Hum. Gene Ther..

[174] Han Z., Conley S.M., Makkia R.S., Cooper M.J., Naash M.I. (2012). DNA nanoparticle-mediated ABCA4 delivery rescues Stargardt dystrophy in mice. J. Clin. Investig..

[175] Kong J., Kim S.R., Binley K., Pata I., Doi K., Mannik J., Zernant-Rajang J., Kan O., Iqball S., Naylor S. (2008). Correction of the disease phenotype in the mouse model of Stargardt disease by lentiviral gene therapy. Gene Ther..

[176] Binley K., Widdowson P., Loader J., Kelleher M., Iqball S., Ferrige G., De Belin J., Carlucci M., Angell-Manning D., Hurst F. (2013). Transduction of photoreceptors with equine infectious anemia virus lentiviral vectors: Safety and biodistribution of StarGen for Stargardt disease. Investig. Ophthalmol. Vis. Sci..

[177] Dyka F.M., Molday L.L., Chiodo V.A., Molday R.S., Hauswirth W.W. (2019). Dual ABCA4-AAV Vector Treatment Reduces Pathogenic Retinal A2E Accumulation in a Mouse Model of Autosomal Recessive Stargardt Disease. Hum. Gene Ther..

[178] Trapani I., Colella P., Sommella A., Iodice C., Cesi G., De Simone S., Marrocco E., Rossi S., Giunti M., Palfi A. (2014). Effective delivery of large genes to the retina by dual AAV vectors. EMBO Mol. Med..

[179] Colella P., Trapani I., Cesi G., Sommella A., Manfredi A., Puppo A., Iodice C., Rossi S., Simonelli F., Giunti M. (2014). Efficient gene delivery to the cone-enriched pig retina by dual AAV vectors. Gene Ther..

[180] Planul A., Dalkara D. (2017). Vectors and Gene Delivery to the Retina. Annu. Rev. Vis. Sci..

[181] Glover D.J., Lipps H.J., Jans D.A. (2005). Towards safe, non-viral therapeutic gene expression in humans. Nat. Rev. Genet..

[182] Sun D., Schur R.M., Sears A.E., Gao S.-Q., Vaidya A., Sun W., Maeda A., Kern T., Palczewski K., Lu Z.-R. (2020). Non-viral Gene Therapy for Stargardt Disease with ECO/pRHO-ABCA4 Self-Assembled Nanoparticles. Mol. Ther..

[183] Han Z., Conley S.M., Naash M.I. (2014). Gene therapy for Stargardt disease associated with ABCA4 gene. Adv. Exp. Med. Biol..

[184] Hu M.L., Edwards T.L., O’Hare F., Hickey D.G., Wang J.H., Liu Z., Ayton L.N. (2021). Gene therapy for inherited retinal diseases: Progress and possibilities. Clin. Exp. Optom..

[185] Semple-Rowland S.L., Berry J. (2014). Use of lentiviral vectors to deliver and express bicistronic transgenes in developing chicken embryos. Methods.

[186] Trapani I., Puppo A., Auricchio A. (2014). Vector platforms for gene therapy of inherited retinopathies. Prog. Retin. Eye Res..

[187] Dyka F.M., Boye S.L., Chiodo V.A., Hauswirth W.W., Boye S.E. (2014). Dual adeno-associated virus vectors result in efficient in vitro and in vivo expression of an oversized gene, MYO7A. Hum. Gene Ther. Methods.

[188] Trapani I., Toriello E., De Simone S., Colella P., Iodice C., Polishchuk E.V., Sommella A., Colecchi L., Rossi S., Simonelli F. (2015). Improved dual AAV vectors with reduced expression of truncated proteins are safe and effective in the retina of a mouse model of Stargardt disease. Hum. Mol. Genet..

[189] Issa P.C., MacLaren R.E. (2012). Non-viral retinal gene therapy: A review. Clin. Exp. Ophthalmol..

[190] Shen X., Corey D.R. (2018). Chemistry, mechanism and clinical status of antisense oligonucleotides and duplex RNAs. Nucleic Acids Res..

[191] Duijkers L., Van den Born L.I., Neidhardt J., Bax N.M., Pierrache L.H.M., Klevering B.J., Collin R.W.J., Garanto A. (2018). Antisense Oligonucleotide-Based Splicing Correction in Individuals with Leber Congenital Amaurosis due to Compound Heterozygosity for the c.2991+1655A>G Mutation in CEP290. Int. J. Mol. Sci..

[192] Cideciyan A.V., Jacobson S.G., Drack A.V., Ho A.C., Charng J., Garafalo A.V., Roman A.J., Sumaroka A., Han I.C., Hochstedler M.D. (2019). Effect of an intravitreal antisense oligonucleotide on vision in Leber congenital amaurosis due to a photoreceptor cilium defect. Nat. Med..

[193] Slijkerman R.W., Vaché C., Dona M., García-García G., Claustres M., Hetterschijt L., Peters T.A., Hartel B.P., Pennings R.J., Millan J.M. (2016). Antisense Oligonucleotide-based Splice Correction for USH2A-associated Retinal Degeneration Caused by a Frequent Deep-intronic Mutation. Mol. Ther. Nucleic Acids.

[194] Bonifert T., Menendez I.G., Battke F., Theurer Y., Synofzik M., Schöls L., Wissinger B. (2016). Antisense Oligonucleotide Mediated Splice Correction of a Deep Intronic Mutation in OPA1. Mol. Ther. Nucleic Acids.

[195] Bauwens M., Garanto A., Sangermano R., Naessens S., Weisschuh N., De Zaeytijd J., Khan M., Sadler F., Balikova I., Van Cauwenbergh C. (2019). ABCA4-associated disease as a model for missing heritability in autosomal recessive disorders: Novel noncoding splice, cis-regulatory, structural, and recurrent hypomorphic variants. Genet. Med..

[196] Garanto A., Duijkers L., Tomkiewicz T.Z., Collin R.W.J. (2019). Antisense Oligonucleotide Screening to Optimize the Rescue of the Splicing Defect Caused by the Recurrent Deep-Intronic ABCA4 Variant c.4539+2001G>A in Stargardt Disease. Genes.

[197] Khan M., Arno G., Fakin A., Parfitt D.A., Dhooge P.P.A., Albert S., Bax N.M., Duijkers L., Niblock M., Hau K.L. (2020). Detailed Phenotyping and Therapeutic Strategies for Intronic ABCA4 Variants in Stargardt Disease. Mol. Ther. Nucleic Acids.

[198] Schwartz S.D., Regillo C.D., Lam B.L., Eliott D., Rosenfeld P.J., Gregori N.Z., Hubschman J.-P., Davis J.L., Heilwell G., Spirn M. (2015). Human embryonic stem cell-derived retinal pigment epithelium in patients with age-related macular degeneration and Stargardt’s macular dystrophy: Follow-up of two open-label phase 1/2 studies. Lancet.

[199] Song W.K., Park K.-M., Kim H.-J., Lee J.H., Choi J., Chong S.Y., Shim S.H., Del Priore L.V., Lanza R. (2015). Treatment of macular degeneration using embryonic stem cell-derived retinal pigment epithelium: Preliminary results in Asian patients. Stem Cell Rep..

[200] Mehat M.S., Sundaram V., Ripamonti C., Robson A.G., Smith A.J., Borooah S., Robinson M., Rosenthal A.N., Innes W., Weleber R.G. (2018). Transplantation of Human Embryonic Stem Cell-Derived Retinal Pigment Epithelial Cells in Macular Degeneration. Ophthalmology.

[201] Sung Y., Lee M.J., Choi J., Jung S.Y., Chong S.Y., Sung J.H., Shim S.H., Song W.K. (2020). Long-term safety and tolerability of subretinal transplantation of embryonic stem cell-derived retinal pigment epithelium in Asian Stargardt disease patients. Br. J. Ophthalmol..

[202] Oner A., Gonen Z.B., Sevim D.G., Kahraman N.S., Unlu M. (2018). Suprachoroidal Adipose Tissue-Derived Mesenchymal Stem Cell Implantation in Patients with Dry-Type Age-Related Macular Degeneration and Stargardt’s Macular Dystrophy: 6-Month Follow-Up Results of a Phase 2 Study. Cell. Reprogram..

[203] Kaufman Y., Ma L., Washington I. (2011). Deuterium enrichment of vitamin A at the C20 position slows the formation of detrimental vitamin A dimers in wild-type rodents. J. Biol. Chem..

[204] Issa P.C., Barnard A.R., Herrmann P., Washington I., MacLaren R.E. (2015). Rescue of the Stargardt phenotype in *Abca4* knockout mice through inhibition of vitamin A dimerization. Proc. Natl. Acad. Sci. USA.

[205] Ma L., Kaufman Y., Zhang J., Washington I. (2011). C20-D3-vitamin A slows lipofuscin accumulation and electrophysiological retinal degeneration in a mouse model of Stargardt disease. J. Biol. Chem..

[206] Radu R.A., Han Y., Bui T.V., Nusinowitz S., Bok D., Lichter J., Widder K., Travis G.H., Mata N.L. (2005). Reductions in Serum Vitamin A Arrest Accumulation of Toxic Retinal Fluorophores: A Potential Therapy for Treatment of Lipofuscin-Based Retinal Diseases. Investig. Ophthalmol. Vis. Sci..

[207] Dobri N., Qin Q., Kong J., Yamamoto K., Liu Z., Moiseyev G., Ma J.-x., Allikmets R., Sparrow J.R., Petrukhin K. (2013). A1120, a Nonretinoid RBP4 Antagonist, Inhibits Formation of Cytotoxic Bisretinoids in the Animal Model of Enhanced Retinal Lipofuscinogenesis. Investig. Ophthalmol. Vis. Sci..

[208] Bavik C., Henry S.H., Zhang Y., Mitts K., McGinn T., Budzynski E., Pashko A., Lieu K.L., Zhong S., Blumberg B. (2015). Visual cycle modulation as an approach toward preservation of retinal integrity. PLoS ONE.

[209] Kubota R., Birch D.G., Gregory J.K., Koester J.M. (2020). Randomised study evaluating the pharmacodynamics of emixustat hydrochloride in subjects with macular atrophy secondary to Stargardt disease. Br. J. Ophthalmol..

[210] Sieving P.A., Chaudhry P., Kondo M., Provenzano M., Wu D., Carlson T.J., Bush R.A., Thompson D.A. (2001). Inhibition of the visual cycle in vivo by 13-cis retinoic acid protects from light damage and provides a mechanism for night blindness in isotretinoin therapy. Proc. Natl. Acad. Sci. USA.

[211] Radu R.A., Mata N.L., Nusinowitz S., Liu X., Sieving P.A., Travis G.H. (2003). Treatment with isotretinoin inhibits lipofuscin accumulation in a mouse model of recessive Stargardt’s macular degeneration. Proc. Natl. Acad. Sci. USA.

[212] Piccardi M., Fadda A., Martelli F., Marangoni D., Magli A., Minnella A.M., Bertelli M., Di Marco S., Bisti S., Falsini B. (2019). Antioxidant Saffron and Central Retinal Function in ABCA4-Related Stargardt Macular Dystrophy. Nutrients.

[213] Liu Q., Sabirzhanova I., Bergbower E.A.S., Yanda M., Guggino W.G., Cebotaru L. (2019). The CFTR Corrector, VX-809 (Lumacaftor), Rescues ABCA4 Trafficking Mutants: A Potential Treatment for Stargardt Disease. Cell. Physiol. Biochem..

[214] Maeda A., Golczak M., Chen Y., Okano K., Kohno H., Shiose S., Ishikawa K., Harte W., Palczewska G., Maeda T. (2011). Primary amines protect against retinal degeneration in mouse models of retinopathies. Nat. Chem. Biol..

[215] Lu W., Campagno K.E., Tso H.Y., Cenaj A., Laties A.M., Carlsson L.G., Mitchell C.H. (2019). Oral Delivery of the P2Y12 Receptor Antagonist Ticagrelor Prevents Loss of Photoreceptors in an ABCA4-/- Mouse Model of Retinal Degeneration. Investig. Ophthalmol. Vis. Sci..

[216] Lu W., Gómez N.M., Lim J.C., Guha S., O’Brien-Jenkins A., Coffey E.E., Campagno K.E., McCaughey S.A., Laties A.M., Carlsson L.G. (2018). The P2Y(12) Receptor Antagonist Ticagrelor Reduces Lysosomal pH and Autofluorescence in Retinal Pigmented Epithelial Cells From the ABCA4^-/-^ Mouse Model of Retinal Degeneration. Front. Pharmacol..

[217] Julien S., Schraermeyer U. (2012). Lipofuscin can be eliminated from the retinal pigment epithelium of monkeys. Neurobiol. Aging.

[218] Julien-Schraermeyer S., Illing B., Tschulakow A., Taubitz T., Guezguez J., Burnet M., Schraermeyer U. (2020). Penetration, distribution, and elimination of remofuscin/soraprazan in Stargardt mouse eyes following a single intravitreal injection using pharmacokinetics and transmission electron microscopic autoradiography: Implication for the local treatment of Stargardt’s disease and dry age-related macular degeneration. Pharmacol. Res. Perspect..

[219] Cao S., Wang J.C.C., Gao J., Wong M., To E., White V.A., Cui J.Z., Matsubara J.A. (2016). CFH Y402H polymorphism and the complement activation product C5a: Effects on NF-κB activation and inflammasome gene regulation. Br. J. Ophthalmol..

[220] Csaky K.G., Bok D., Radu R.A., Sadda S.R. (2018). Complement C5 Inhibition as a Potential Treatment for Autosomal Recessive Stargardt Disease (STGD1): Design of a Clinical Trial Assessing a Novel Treatment and Primary Outcome Measure. Investig. Ophthalmol. Vis. Sci..

[221] MacDonald I.M., Sieving P.A. (2018). Investigation of the effect of dietary docosahexaenoic acid (DHA) supplementation on macular function in subjects with autosomal recessive Stargardt macular dystrophy. Ophthalmic Genet..

[222] Cornish K.S., Ho J., Downes S., Scott N.W., Bainbridge J., Lois N. (2017). The Epidemiology of Stargardt Disease in the United Kingdom. Ophthalmol. Retin..

[223] Cideciyan A.V., Swider M., Aleman T.S., Tsybovsky Y., Schwartz S.B., Windsor E.A.M., Roman A.J., Sumaroka A., Steinberg J.D., Jacobson S.G. (2009). ABCA4 disease progression and a proposed strategy for gene therapy. Hum. Mol. Genet..

[224] Liu F., Lee J., Chen J. (2021). Molecular structures of the eukaryotic retinal importer ABCA4. Elife.

